# Influence of QuEChERS modifications on recovery and matrix effect during the multi-residue pesticide analysis in soil by GC/MS/MS and GC/ECD/NPD

**DOI:** 10.1007/s11356-016-8334-1

**Published:** 2017-01-16

**Authors:** Bożena Łozowicka, Ewa Rutkowska, Magdalena Jankowska

**Affiliations:** Plant Protection Institute - National Research Institute, Laboratory of Pesticide Residues, Chelmonskiego 22, Postal code: 15-195 Bialystok, Poland

**Keywords:** Pesticide, Soil, Optimization, Multi-residue method, QuEChERS, Gas chromatography

## Abstract

A QuEChERS extraction followed by GC/MS/MS and GC-μECD/NPD for 216 pesticide and metabolites determination in soil simultaneously were developed and compared. Volume of water, volume and polarity of solvent, and cleanup sorbents (C18, GCB, PSA) were optimized. The QuEChERS with and without purification step were applied to estimate effectiveness of the method. The recovery and matrix effect (ME) were critical parameters within each tested procedure. The optimal method without cleanup was validated. Accuracy (expressed as recovery), precision (expressed as RSD), linearity, LOQ, and uncertainty were determined. The recoveries at the three spiking levels using matrix-matched standards ranged between 65 and 116% with RSD ≤17 and 60–112% with RSD ≤18% for MS/MS and μEC/NP, respectively. The LOQ ranged from 0.005–0.01 mg/kg for MS/MS to 0.05 mg/kg for μEC/NP. The ME for most of pesticides resulted in enhancement of the signal and depended on the analyte and detection system: MS/MS showed ME from −25 to 74%, while μEC/NP from −45 to 96%. A principal component analysis was performed to explain the relationships between physicochemical parameters and ME of 216 pesticides. The QuEChERS protocol without the cleanup step is a promising option to make the method less expensive and faster. This methodology was applied in routine analysis of 263 soil samples in which p,p’ DDT was the most frequently detected (23.5% of samples) and pendimethalin with the highest concentration (1.63 mg/kg).

**Electronic supplementary material:**

The online version of this article (doi:10.1007/s11356-016-8334-1) contains supplementary material, which is available to authorized users.

## Introduction

Soil is an important resource of agriculture which has an ability to retain agro-chemicals. Soil contamination causes the presence of xenobiotic chemicals and very varied from industrial activity, improper disposal of waste to agricultural chemicals. The presence of pesticide compounds in soils may have different sources: direct application, accidental spillage, runoff from the surface of plants, or from incorporation of pesticide contaminated plant materials (Rashid et al. 2010). Agricultural soil is a high value component, so its irreversible degradation should be avoided to guarantee its fertility and current and future value.

Soil is a complex and heterogeneous matrix with a porous structure that contains both inorganic (variable percentage of sand, silt, and clay) and natural organic components mainly composed by humic substances (10–15%), lipids, carbohydrates, lignin, flavonoids, pigments, resins and fulvic acids (Pinto et al. 2011). These compounds are characterized by the diverse chemical structure and physicochemical properties, which cause many analytical problems. Therefore, pesticide analysis at low concentration levels in these samples is a very difficult and challenging task.

In the literature, the analytical procedures for the determination of pesticide residues in soil commonly are based on traditional sample preparation methods, such as: liquid solid (LSE) (Durović et al. 2012), solid phase extraction (SPE) (Dąbrowska et al. 2003), ultrasonication in acetone (Harrison et al. 2013), and in soxhlet apparatus extraction (Sanghi and Kannamkumarath 2004). Other methods, such as accelerated solvent (ASE) (Rouvière et al. 2012), dispersive liquid-liquid microextraction (DLLME) (Pastor-Belda et al. 2015), matrix solid phase dispersion (MSPD) (Łozowicka et al. 2012), ultrasonic solvent (USE) (Tor et al. 2006), microwave assisted (MAE) (Guo and Lee 2013; Fuentes et al. 2007), pressurized liquid (PLE) (Martinez Vidal et al. 2010; Masiá et al. 2015), solid phase microextraction (SPME) (Moreno et al. 2006), supercritical fluid extraction (SFE) (Naeeni et al. 2011) have been developed to reduce the amount of reagents and time provided on sample preparation.

Nowadays, in pesticide residue analysis, QuEChERS method (*ang. Ouick, Easy, Cheap, Effective, Rugged and Safe*), developed by Anastassiades et al. (2003), become a very popular technique for different matrix sample preparations such as: cereals (He et al. 2015), fruit and vegetables (Lehotay et al. 2010), honey (Bargańska et al. 2013), tea (Lozano et al. 2012) and tobacco (Łozowicka et al. 2015), because of its simplicity, low cost, amenability to high throughput, and high efficiency with a minimal number of steps. It involves two steps, extraction based on partitioning between an aqueous and an organic layer via salting-out and dispersive SPE for further cleanup using combinations of MgSO_4_ and different sorbents, such as C_18_, primary-secondary amine (PSA), or graphitized carbon (GCB) to remove interfering substances (Anastassiades et al. 2003).

The QuEChERS method has been described to a limited extent for the extraction of wide range of pesticides from soil. The QuEChERS methodology was the first time applied to the extraction of pesticides from soils in 2008 by Lesueur et al. (2008). In that study, the authors compared different extraction methods for 24 pesticides that were commonly reported as soil pollutants in the literature, those belonging to specific classes. Other researchers have applied the QuEChERS for the extraction of the particular classes such as the amide, carbamate, organochlorine, organophosphorus, triazine, triazinone, thiadiazine, and urea. (Asensio-Ramos et al. 2010; Correia-Sá et al. 2012; Li et al. 2012; Fernandes et al. 2013; Mantzos et al. 2013; Masiá et al. 2015).

Gas chromatography (GC) with the variety of sensitive detectors such as electron capture (EC) and nitrogen phosphorus (NP) (Łozowicka et al. 2012), mass spectrometry (MS) (Rouvière et al. 2012; Wu and Hu 2014), tandem mass spectrometry (MS/MS) (Rashid et al. 2010) are techniques usually utilized in pesticide residue analysis in soils. Besides GC, which has some limitation, a perfect complement is high or ultra-high pressure liquid chromatography (HPLC, UHPLC) (Martinez Vidal et al. 2010; Moreno et al. 2006), liquid chromatography–mass spectrometry (LC/MS) (Chen et al. 2010) or tandem mass spectrometry (LC/MS/MS) (Kaczyński et al. 2016).

Despite the continuous appearance of many new analytical methods and instrumental equipments, one of the greatest difficulties in pesticide residue analysis is matrix effect and its unfavorable influence on quantitative and qualitative analyte determination, particularly in the analysis of complex samples. Matrix effect depends on the nature of compounds (molecular size, polarity, thermal stability, volatility, etc.) and the analyte concentration. Numerous methods have been proposed to correct its effects, including the use of analyte protestants (Anastassiades et al. 2013), coated inlet liners, compensation factors, different injection techniques, dilution, internal standards, extensive sample cleanup, GC priming, and labeled internal standards, but the majority method is to perform matrix-matched calibrations (Erney et al. 1997).

Therefore, an existing knowledge needs to be filled (Vera et al. 2013; Bruzzoniti et al. 2014) by finding cheaper and faster method for the simultaneous analysis of pesticides covering a wide range of polarities in complex matrix such as soil that has been carried out. On the results of an analysis, affect interfering substances can be co-extracted with analytes; thus, it is very challenging to determine substances at very low concentration levels. Due to the use in agriculture of diverse classes of pesticides, multi-residue methods are required for the accurate and simultaneous determination of pesticides.

In this paper, the influence of modifications of QuEChERS on the recovery and matrix effect during the analysis of over 50 multiple classes’ of pesticides in soil was reported. An additional objective of the study was to determine and compare the extent and variability of matrix effects of analytes using gas chromatography with different types of detectors. Otherwise, it was attempted to find the correlation between selected physicochemical properties of 216 pesticides including metabolites and matrix effect using a principal component analysis (PCA).

## Material and methods

### Reagents and materials

Acetone, acetonitrile (AcN), and ethyl acetate (EtOAc) were analytical grade and provided for pesticide residue analysis by J.T. Baker (Deventer, The Netherlands). Water was purified by Milli-Q (Millipore, Billerica, MA, USA) system. Water was cooled to temperature about 4 °C. QuEChERS sorbent kits and pouches of salts were purchased from the Agilent Technologies (Santa Clara, CA, USA). The sorbents used in this study were as follows: PSA (primary-secondary amine), C18, GCB (graphitized carbon black), and pouches of salts: magnesium sulfate, sodium chloride, sodium citrate, citric acid disodium salt. Formic acid were supplied by Fluka (98% purity).

Pesticides (purity for all standards >95%) were purchased from Dr. Ehrenstorfer Laboratory (Augsburg, Germany). The triphenyl phosphate (TPP, 20 mg/mL) as the internal standard was obtained from Sigma-Aldrich (Steinheim, Germany). For GC-μECD/NPD analysis, each stock standard solution was prepared at various concentrations (at range 100–250 mg/mL) in acetone and stored in dark below 4 °C (for GC/MS/MS at 100 mg/mL). Standard working solutions of multi-compounds were prepared by dissolving the appropriate amounts of each stock solution in *n*-hexane/acetone (9:1, *v*/*v*) mixture. The stock and working solutions were stored in completely filled vials, closed with parafilm at −20 °C until the time of analysis.

### Soil samples

Blank soil samples previously check for the presence of pesticides, for the method optimization and validation were used. Soils were collected with a stainless steel scoop in depth between 0 and 20 cm from the field located from the vicinity of Bialystok (53°07′ N latitude and 23°09′ E). The soil samples were stored in PE bags at 4 °C away from light. Soil samples were homogenized, sieved (2-mm mesh) and air-dried at room temperature before their use. The physicochemical characteristics of soil are the following: textural class—loamy sand, organic matter 1.45%, pH 6.6, % silt 22.45 (0.002–0.05 mm), % sand 75.32 (0.05–2 mm), and % clay 2.43 (<0.002 mm).

### Sample preparation

Representative portions of soil (500 g) was air-dried at about 40 °C and then sieved through a mesh with a grain size of 2 mm. They were stored at room temperature until analysis.

Five grams of homogenized soil sample and 10 mL of cold purified water in a 50 mL polypropylene centrifuge tube were hand shaken for 1 min to hydrate the samples and allowed to stand for 10 min. Ten milliliters of 1% formic acid in acetonitrile and 100 μL of internal standard solution TPP (in the case of GC/MS/MS) were added and the sample was vortexed for 7 min. A salt mixture, 4 g MgSO_4_, 1 g NaCl, 1 g trisodium citrate dihydrate (Na_3_C_6_H_5_O_7_•2H_2_O), and 0.5 g disodium hydrogen citrate sesquehydrate (Na_2_HC_6_H_5_O_7_•1.5H_2_O), was added. The tube was immediately shaken for 1 min to prevent formation of crystalline agglomerates during MgSO_4_ hydration and vortexed for 5 min at 4500 rpm. The tube was placed in the −60 °C freezer for 30 min and let the supernatant reach room temperature. Two milliliters of extract were transferred into a flask and acidified with 20 μL of 1% formic acid in acetonitrile. Two droplets of dodecane were added. The extract was evaporated at 40 °C in a rotary evaporator to near dryness. The residue was dissolved in 2 mL *n*-hexane/acetone (9:1, *v*/*v*) and was filtered through a 0.45 μm nylon filter to an autosampler vial and subsequently analyzed via GC/MS/MS.

Vortex-Mixer (Velp Scientifica, Usmate, Italy) and Rotina 420R (Hettich, Tuttlingen, Germany) were used in sample extraction.

### GC/MS/MS analysis

The analysis was performed by GC/MS/MS: an Agilent 7890A GC system (Agilent Technologies, Palo alto, CA, USA) was equipped with an Agilent 7693 autosampler and was coupled to a triple quadrupole mass spectrometer 7000B (Agilent Technologies) and operated in electron ionization mode (EI −70 eV). Splitless injection of a 2-μL sample was separated by an HP-5 MS capillary column ((5%-phenyl)-methylpolysiloxane; 30 m × 0.25 mm ID and film thickness of 0.25 μm; Agilent Technologies). The oven temperature was programmed as follows: 70 °C (2 min hold) to 150 °C at a rate 25 °C/min^−1^, increased to 200 °C at 3 °C/ min^−1^, and finally to 280 °C at 8 °C/min^−1^ and held for 10 min. Helium (99.9998% purity) was used as the carrier gas at a constant flow rate of 2.1 mL/min^−1^. The total running time was 41.88 min. The temperatures of the transfer line, the ion source, first quadrupole, and second quadrupole were 280, 300, 180, and 180 °C, respectively. Helium (99.9998% purity) and nitrogen (99.9998% purity) were collision gases at a flow rate of 2.25 and 1.5 mL/min^−1^, respectively. MassHunter quantitative analysis software (version B.06.00) (Agilent Technologies) was used for data processing. MRM transitions and other acquisition parameters can be found in Table S1.

### GC-μECD/NPD analysis

Pesticides were analyzed by using an Agilent (Waldbronn, Germany) model 7890 A gas chromatograph (GC) equipped with micro-electron capture (μEC) and nitrogen phosphorus (NP) detectors. A capillary column HP-5 ((5%-phenyl)-methylpolysiloxane; 30 m × 0.32 mm ID and film thickness 0.25 μm, Agilent Technologies) were used. Chemstation quantitative analysis software (version A.10.2) (Agilent Technologies) was used for data processing. The injector and detector temperature were set at 210 and 300 °C, respectively. The oven temperature was programmed as follows: 120 to 190 °C at a rate 16 °C min^−1^, increased to 230 °C at 8 °C min^−1^, and finally to 285 °C at 18 °C min^−1^ and held for 10 min. Helium (99.9998% purity) was used as carrier gas at a flow rate of 3.0 mL min^−1^. Nitrogen (99.9998% purity) as a make-up gas at a flow rate of 57 mL min^−1^ (for EC) and 8 mL min^−1^ (for NP) was used. Hydrogen (99.9998% purity) and air (99.9998% purity) (for NP) gas flows were set at 3.0 and 60 mL min^−1^. Two microliters of the sample extract was injected in splitless mode (purge-off time 2 min). Total time of analysis is 25 min.

### Method validation

Soil samples, free of pesticides, were used for this study. The method validation was performed using the following parameters: accuracy (expressed as recovery), precision (expressed as RSD), linearity (expressed as *R* ^2^), limit of quantification (LOQ), and uncertainty according to the European Union guideline SANCO (SANCO 2013).

The method accuracy and precision were evaluated by performing recovery studies. Precision was expressed as relative standard deviation (%RSD). Accuracy was expressed as and recovery. Three different levels have to be analyzed (LOQ, 10 × LOQ, 100 × LOQ) with five replicates for each level on five different days. After homogenization, matrix blanks were spiked with the pesticide standard mixture and equilibrated for 30 min at room temperature prior to QuEChERS extraction to allow the pesticides to be incorporated into the soil matrix.

Linearity was studied by analyzing matrix-matched standards at five concentration levels. The range of analyzed concentrations was within the range of LOQ to 100 × LOQ. The LOQ for each pesticide was defined as the lowest spiking level meeting the requirement of recovery and RSD for different fortification levels. Expanded measurement uncertainties were estimated using a “top-down” empirical model according to the data obtained in the validation study (coverage factor *k* = 2, confidence level 95%).

For special group of pesticides (pyrethroid insecticides), the quantification of these compounds was performed by summing the peak areas of their isomers. They contain two or three chiral centers, making them a family of pesticides with stereoisomers. Therefore, multiple peaks were observed for several of the pyrethroids, corresponding to the separation of their diastereoisomers. Deltamethrin, difenoconazole, dimethomorph, esfenvalerate/fenvalerate, *lambda* cyhalothrin, permethrin, *tau* fluvalinate, and tetramethrin were resolved using two peaks, while four peaks were observed for cypermethrin and cyfluthrin (Li et al. 2012).

### Matrix effect and process efficiency

Initially, all of the procedures were evaluated in terms of ME, by comparison between the areas of standard in the extract and the standard in the solvent, as shows equation: ME (%) = (area of the standard in matrix/area of the standard in the solvent) × 100. The ME near to 100% indicated no influence from the matrix, while out of the range 80–120% showed significant matrix effect. For the validated method, using procedure without cleanup, the ME was calculated as follows: ME (%) = [(slope in matrix/slope in solvent)−1] × 100. Negative values of matrix effects signify suppression of the signal, and positive values signify enhancement. For better understanding of the results, the values were categorized into three groups: (i) soft matrix effect <±20%, (ii) medium >±20 and <±50%, and (iii) strong >±50%. Values <20% indicated no ME or its insignificance, and values >20% were considered as a high ME.

The process efficiency (PE) evaluates the overall performance of the extraction method. The PE was calculated as follows: PE = (*R* × ME)/100. PE values near 100% indicated recoveries and low matrix effect (Arias et al. 2014).

### Statistical analysis

PCA was performed to explain the relationships between physicochemical parameters and matrix effect of 216 pesticides and metabolites in complex soil matrices. Data were statistically evaluated by PCA using Statistica version 11.0 software (StatSoft).

## Results and discussion

### Comparison of soil sample preparation and choice of the optimum method

This study presents modification of one of the most widely described multi-residue methodologies —QuEChERS approach, which has many advantages including speed, cost, and ease of use; good performance characteristics; and wide applicability range (matrices and analytes).

Volume of water, solvent volume and polarity, and cleanup sorbents (C18, GCB, PSA), which are parameters affecting the extraction efficiency, were optimized to get the “cleanest” matrix. The QuEChERS with and without purification step were applied to estimate effectiveness of the method.

The first step for modification is based on added water to dry soil samples before extraction. Soil samples belong to matrices with low-moisture matrices. The original QuEChERS method was designed for samples with more than 75% moisture and for products with a water content lower than 25%; the QuEChERS method has been modified (Cajka et al. 2012). According to Cajka et al. (2012), adding water to the sample is a key to achieve maximum extraction yield and accurate results. Therefore, choice of appropriate volume of cold water was tested. Additionally, cold water that is used to compensate the heat generated when magnesium sulfate is added to sample during extraction (an exothermic reaction); this helps to protect heat-sensitive pesticides. The tested water dosages were 5, 7.5, and 10 mL. It was not possible to use the same amount of water (5 mL) as sample (5 g) because of the complete absorption of the whole volume of water by the soil. For about 40% of the tested compound better recoveries were obtained when the soil was hydrated with 10 mL of water for 5 g soil. Some authors have tested different ratios (sample:water) and compared the recoveries obtained with various volumes of water addition (Radish et al. 2010; Correia-Sá et al. 2012; Fernandes et al. 2013). The results confirmed the importance of the hydration step for the success extraction of the analytes.

The proposed residue analysis covered the wide range of pesticides; therefore, the appropriate conditions of extraction and isolation must be ensured.

One of the critical steps and the most important parameters to optimize is choice of extraction solvent. In extraction of target pesticides from solid sample, the extraction solvent must be characterized by a high dissolving ability for pesticides and good permeability into the matrix, especially for dry samples (Kolberg et al. 2011). Therefore, three solvents for extraction (sorted in order of increasing polarity index, PI): EtOAc (PI 4.4), acetone (PI 5.1), and AcN (PI 5.8) were used. These solvents were chosen, because they are commonly used for multi-residue analysis for a wide range of pesticides in different food matrices such as fruit and vegetables (Mol et al. 2007), olive (Moreno López et al. 2014), sugar beets, and beet molasses (Łozowicka et al. 2016). The extraction of acetone or EtOAc gave similar recoveries and 25% of all tested compounds have unsatisfactory values. More polar compounds (e.g., azoxystrobin 69 → 106%, dicrotophos 65 → 85%, methamidophos 59 → 79%, propoxur 72 → 85%) showed recovery increase when the AcN was used in comparison to acetone/EtOAc. Acetonitrile was selected because it yielded acceptable extraction efficiency in a wide range of pesticides. Finally, for extraction, 10 mL of 1% formic acid in acetonitrile was added.

Additionally, addition of the internal standard (TPP) to the samples after the extraction solvent allows to control the entire analytical process what contributes to minimization of the error generated in the multiple steps and improves precision and accuracy.

Pesticides such as base- and acid-sensitive which require special pH were within the scope of this analysis. Therefore, pH is a very important parameter in the stability of several base-sensitive, e.g., captan (76%), dichlofluanid (65%), dicofol (85%), folpet (68%), and tolylfluanid (68%) and it is also critical for acid-sensitive pesticides, e.g., amitraz (75%) and carbosulfan (73%). Therefore, by adding the citrate buffering salts, the samples obtained pH values between 5.0 and 5.5. This pH range was a compromise, between the quantitative extraction and protection of alkali and acid-labile compounds.

The most important task of extraction is not only transferring interested analytes from the matrix to the extraction solvent but also reducing the co-extracted components of matrix as far as possible, because this background may negatively affect the ruggedness of the GC analysis. Therefore, the parts of co-eluting compounds were separated from the extracts to a large extent by putting them in the freezer (−60 °C) for 30 min.

The need for further purification step was examined and the results were compared to those without cleanup. Cleanup step was necessary in preparation of complex matrices such as soil to reduce interferences, improve quantification, and do not disturb the signal on the chromatographic system.

Therefore, in this work efficiency of removal of impurities by three kinds of d-SPE adsorbents was tested. Octadecylsilane (C18) is a nonpolar sorbent that effectively traps and removes trace amounts of lipids, starch, sugar, and other interferences as humic substances. Primary-secondary amine (PSA) is a weak anion exchange sorbent that removes sugars a fatty and other acids. Graphitize carbon black (GCB) is used for removal of pigments.

To remove residual water, PSA sorbent with anhydrous magnesium sulfate (MgSO_4_) and their mixtures with GCB or/and C_18_ were applied. The sorbents were the following:

1. 20 mg anhydrous MgSO_4_ + 10 mg PSA,
2. 20 mg anhydrous MgSO_4_ + 25 mg PSA + 25 mg C18;
3. 20 mg anhydrous MgSO_4_ + 25 mg PSA + 2.5 mg GCB;
4. 20 mg anhydrous MgSO_4_ + 30 mg PSA + 25 mg C18 + 2.5 mg GCB.

PSA was used in each variant. PSA is the most common sorbent used and can act both as a polar phase and weak anion exchanger with the ability to remove a lot of matrix co-extractives (Kinsella et al. 2009). Addition to the extract of PSA increases the pH of the extracts, reaching values above 8. This compromises the stability of base-sensitive pesticides (e.g., captan, chlorthalonil, and folpet). On the other hand, degradation of acid-labile pesticides (e.g., amitraz, carbosulfan) was reduced sufficiently by acidifying the extracts quickly up to pH ~5 by adding 1% formic acid in acetonitrile. This step allowed storing the extracts for several days at room temperature without the occurrence of unacceptable losses of most pesticides, particularly for acid-labile pesticides.

The useful parameter to assess the effectiveness of the purification step is recovery and matrix effect (ME). The European Union guideline SANCO (SANCO 2013) as the acceptance criteria of the validation parameters of the method was adopted to procedure, according to which the average recovery should be in the range 70–120% with RSD less or equal 20%. A practical default range of 60–140% may be used for individual recoveries in routine analysis. Recovery and matrix effect were studied using MS/MS and μECD/NPD detection.

**Fig. 1:**
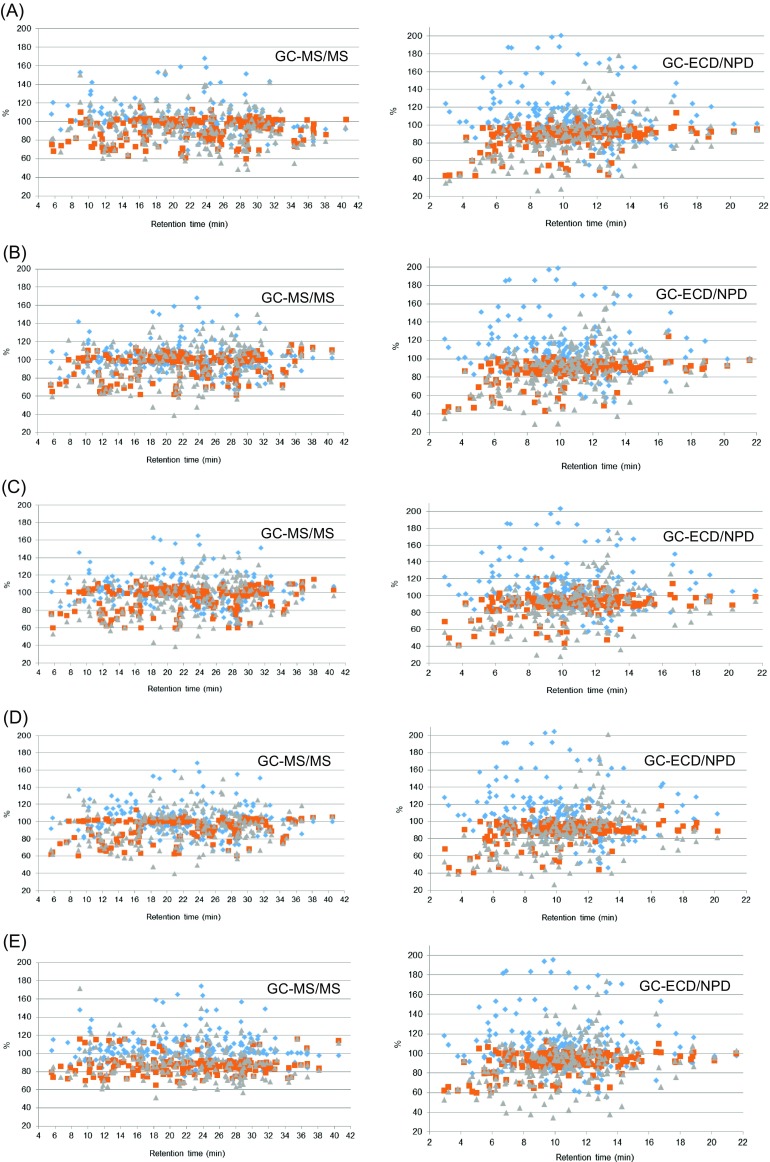
Recoveries, matrix effects, and process efficiency of pesticides from varying d-SPE cleanup conditions using GC/MS/MS and GC-μECD/NPD: **a** MgSO_4_ + PSA, **b** MgSO_4_ + PSA + C18, **c** MgSO_4_ + PSA + GCB, **d** MgSO_4_ + PSA + C18 + GCB, and **e** without cleanup

In the first variant (A), as is presented in Fig. 1a, the number of pesticides with satisfactory recovery was obtained using PSA sorbents (95%, 91% analysis by GC/MS/MS and GC-μECD/NPD, respectively) as well as in combination with C18 (variant B). This sorbent also ensured the best matrix effect values. Matrix effect in the range 80–120% showed 77% of tested compounds in MS/MS and μECD/NPD detection.

The use of nonpolar sorbents such as the octadecyl (C18) with combination with PSA (variant B) during cleanup gave satisfactory recoveries for the most of the target compounds analyzed by GC/MS/MS and GC-μECD/NPD (95%, 91% of tested compound, respectively) (Fig. 1b). For this combination recoveries did not reach the expected range 60–140% for 12 substances and 25% of analyzed pesticides showed ME outside the acceptable range.

In the variant C and D based on GCB addition, about 5% of pesticides had very low recovery values (Fig. 1c, d). Fourteen of the compounds had unacceptable recoveries when the combination with PSA and 11 with PSA + C_18_ was used. The use of GCB retained some planar pesticides (e.g., captan, chlorothalonil, dichlobenil, dichlorvos, dichlofluanid, folpet, methacrifos, imazalil, thiabendazole, and tolylfluanid) and thus sorbent was not used in the further cleanup. In addition, the matrix effects for about 23% of pesticides (above 120%) signified strong enhancement of the chromatographic signal determined by GC/MS/MS and GC-μECD/NPD.

Similarly, the effectiveness of QuEChERS method without purification was estimated.

The procedure without the cleanup step gave very good recoveries (70–120%) for almost all tested compounds expect five pesticides using GC/MS/MS and 17 using GC-μECD/NPD with recoveries between 60 and 69% (RSD 1–17% and 1–19%, respectively) (Fig. 1e). It is evident that matrix effects of the QuEChERS method without cleanup step are generally less pronounced (%ME closer to 100%) than matrix effects for the QuEChERS method when sorbents are used. Only 13% tested substances showed ME values insignificantly outside the range of 80–120%.

The use of different sorbents did not have a significant influence on the recovery of pesticides from the extracts.

Similarly, Caldas et al. (2011) and Wang et al. (2012) studied PSA and C_18_ sorbents for soil sample cleanup and proved that they did not have a significant influence on the purification and recovery of analytes from the extract.

### Process efficiency

Another useful parameter in assessing the effectiveness of cleaning by PSA and its combinations of a purification step and without cleanup step and help to choice the most appropriate method was process efficiency (PE) introduced by Varga et al. (2011). PE was evaluated in order to obtain a direct relationship between the recovery of the analytes and matrix effect.

PE was calculated and compared for QuEChERS method without and with cleanup step for all tested sorbents (Fig. 1). Generally, the signal enrichment due to the ME (e.g., azaconazole, isoprocarb, imazalil) usually increases the PE (e.g., bifenazat, fipronil, iprodione). QuEChERS method without purification had more compounds within the range 80–120% and satisfactory results were obtained for the 64% of tested pesticides.

Basing on optimal parameters such as recovery, matrix effect and summering process efficiency QuEChERS method without cleanup was chosen as the most efficient method. Additionally, an advantage of this procedure without cleanup is more practical due to consumption of less solvent and ewer reagents in comparison with method including purification step.

### Method validation

The optimized analytical method without cleanup step for 216 pesticides and metabolites in soil using MS/MS and μEC/NPD detection was evaluated. Different parameters such as accuracy (expressed as recovery), precision (expressed as RSD), linearity (expressed as *R* ^2^), LOQ, and uncertainty were determined. Validation parameters obtained in this study are shown in Supplementary data Table S2.

Recovery and precision of the proposed method for all pesticides at three spiking levels (LOQ, 10 × LOQ, 100 × LOQ mg/kg) in five replicates were performed. In the case of MS/MS detection, the recoveries for almost all pesticides (without five 65–69%) were satisfactory and ranged from 71 to 120% (RSD 1–17%). Contrary to μECD/NPD detection, 17 analytes which showed 60–69% with acceptable other validation parameters (RSD 1–18%). In both systems of detection, for some planar compounds such as captan, dichlofluanid, folpet, thiabendazole, and tolylfluanid, low recoveries between 63 and 69% were obtained. For instance, captan and folpet are prone to degradation during sample preparation and GC injection; thus, it could be the possible reason for their relatively poor analytical performance. However, other validation parameters were satisfactory (RSDs of <20% were acceptable) (SANCO 2013). Moreover, dichlobenil, dichlorvos, diphenylamine, and methamidophos analyzed by selective detectors μECD/NPD showed recoveries in the range 60–66%. In both MS/MS and μECD/NPD detection, general tendency of higher RSD values at low spiking concentrations equal LOQ was observed.

Linearity was assessed using matrix-matched calibration solutions at five concentration levels, LOQ, 2 × LOQ, 10 × LOQ, 50 × LOQ, and 100 × LOQ for each pesticide. LOQ was set at the lowest spiking concentration and was within the range 0.005–0.01 mg/kg for MS/MS and 0.005–0.05 mg/kg for μECD/NPD, showing that detector MS/MS used in analysis was more sensitive than μECD/NPD. Linearity of both system of detector response was similar and found for all pesticides at concentrations within the test intervals, with the linear regression coefficients (*R* ^2^) higher than 0.99.

The expanded measurement uncertainties were established using a “top-down” empirical model and their values ranged from 10 to 28% and from 16 to 30% (coverage factor *k* = 2, confidence level 95%) by using MS/MS and μECD/NPD, respectively. This is distinctively less than the maximum threshold value of 50% recommended by European Union guidelines (SANCO 2013), demonstrating suitability of the optimized and validated method.

The proposed instrumental method (μGC-ECD/NPD) allowed for the determination of pesticides in soil by GC with two selective detectors functioning simultaneously. In the presented work, we used configuration with a “Y” piece at the end of the GC column in order to divide the flux at the end of the GC column into two branches of equal flow (one to the NPD and the other to the ECD), thus allowing pesticides of different nature to be quantified in the same run; 180 pesticides were detected by the ECD, whereas 179 were analyzed by NPD, although ECD and NPD also provided a discernible signal for 143 of them (Łozowicka et al. 2015).

### Matrix effect

The challenging task in this study was to estimate the variability of matrix effects for 216 representative pesticides and metabolites in soil samples extracted using QuEChERS method without cleanup for MS/MS and μECD/NPD analysis. Matrix interferences are one of the major problems of pesticide residue analysis in different matrices because it can suppress or enhance the chromatographic signals (Kruve et al. 2008, Zhang et al. 2011). These effects may result in low or high analyte recoveries, respectively. This problem may be omitted by preparing matrix-matched standards instead of pure solvent, which was presented in this work.

Matrix effects for almost all pesticides analyzed by MS/MS and μECD/NPD detection exhibited enhancement more common than suppression. It is typical for the GC to observe an enhancement effect resulting from blocking of active column sites by matrix components; thus, more pesticide particles can reach the detector (Anastassiades et al. 2003). In GC/MS/MS, the signal enhancement was observed for 65% analyzed pesticides and in GC-μECD/NPD for 55%. Results from the evaluation of the ME under the optimized QuEChERS conditions MS/MS and μECD/NPD detection are presented in Table S2. However, not all the compounds were equally vulnerable to enhancement. For example, bifenthrin and iprodione eluted with the same retention time but the first compound exhibited 9% (MS/MS) and 13% (GC-μECD/NPD) enhancement whereas the second −25% (GC/MS/MS) and −39% (GC-μECD/NPD) was suppressed.

In the soil extracts analyzed by MS/MS, 87% pesticides exhibited MEs lower than ±20%; 10.6% showed a medium ME with values ranging from −25 to −21% and 21 to 49%, only six pesticides showed a strong ME (bromacyl, dicofol, dimoxystrobin, imazalil, p,p DDE, and thiabendazole). Imazalil showed the greatest ME value of 74%.

Appling μECD/NPD detection for analysis, 74% pesticides showed a soft ME; 36 pesticides had values ranging from −45 to −21% and from 21 to 47% and the remaining 17 pesticides showed a strong ME. Thiabendazole exhibited the greatest signal enhancement of 96%.

**Fig. 2:**
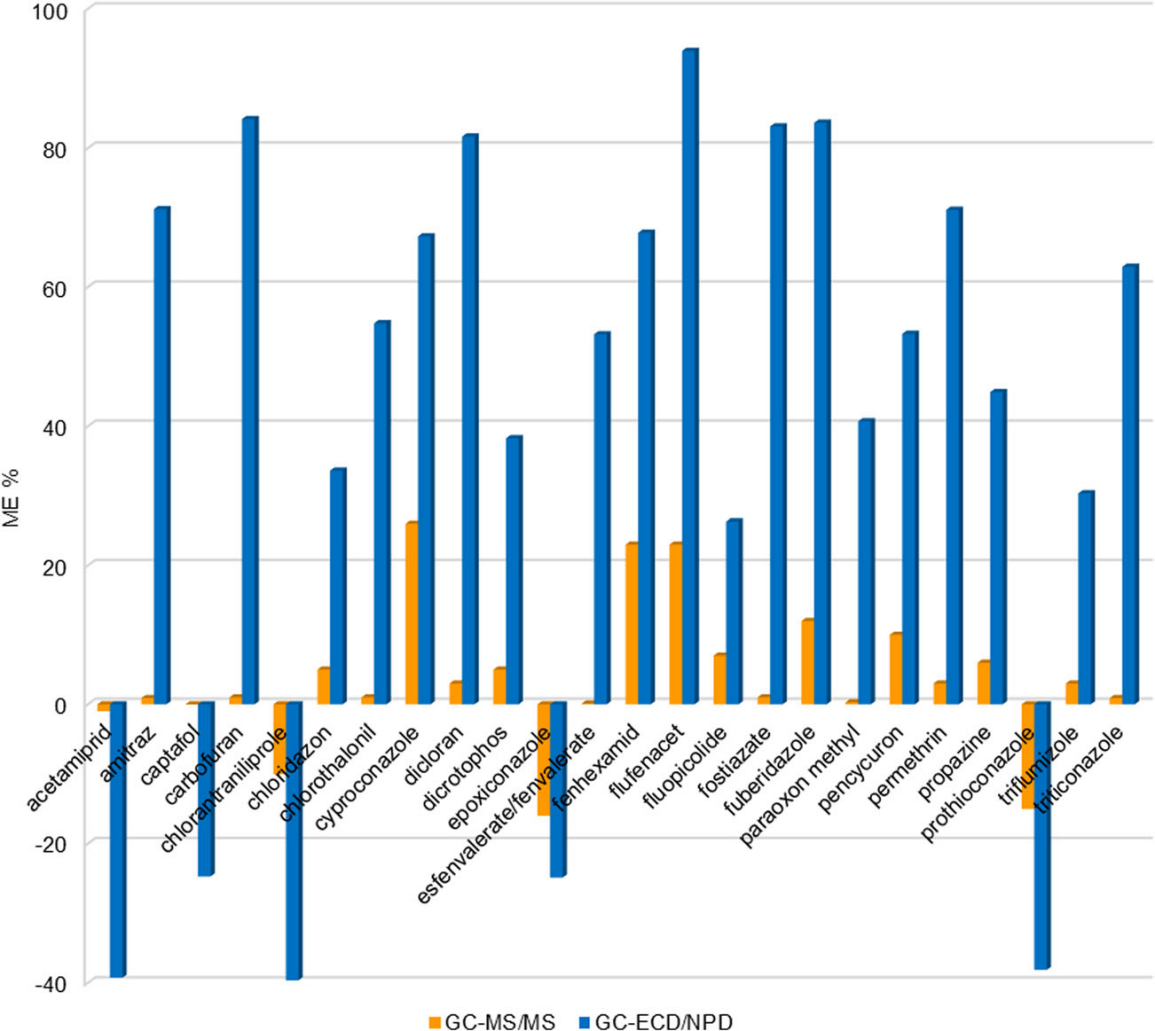
Matrix effect (ME %) for selected pesticides obtained by optimized QuEChERS method

Generally, for MS/MS detection, values of ME were smaller than those found with μECD/NPD. This finding was corresponded for several pesticides including acetamiprid, amitraz, carbofuran, chlorothalonil, cyproconazole, dicloran, esfenvalerate/fenvalerate, fenhexamid, flufenacet, fostiazate, fuberidazole, permethrin, pencycuron, and triticonazole (Fig. 2). Probably, the interferences and background noise were reduced by the use of a more sensitive MS/MS system.

The rule of suppression or enhancement for the most analytes in MS/MS and μEC/NP detection was observed except for the group of 37 compounds (17%, e.g., EPN, endrin, fenarimol, kresoxim-methyl, mevinphos, oksadiksyl, propiconazole, and triazophos (Table S2)).

For instance, alpha, beta, and sulfate endosulfane; dichlorvos; methamidophos; oxyflurofen; and oxamyl showed positive matrix effects, in contrast to bupirimate, dichlobenil, etaconazole, propham, trifloxystrobin with negative matrix effects using MS/MS and μEC/NP detection.

Similarly, other authors also found that ME was a major drawback for quantitative trace determination of pesticides in soil samples, so they used matrix-matched calibrations (Radish et al. 2010; Correia-Sá et al. 2012). Fernandes et al. (2013) observed ME for 12 pesticides (α- and β-HCH, HCB, endrin, o,p̕-DDT, bupirimate, chlorpyrifos, fludioxonil, iprodione, malathion, methiocarb, and pendimetaline) from a group of 36 pesticides. In this study, ME was confirmed only for iprodione among the tested compounds listed above (−25% MS/MS and −39% μECD/NPD). Asensio-Ramos et al. (2010) observed significant ME for 11 pesticides (buprofezin, chlorpyrifos, chlorpyrifos-methyl, diazinon, dimethoate, ethoprofos, fenirothion, malaoxon, malathion, and phosmet) and in this study no ME was achieved for these substances.

In order to better understand the matrix effect in the method without cleanup, correlations between selected parameters (molecular mass, log P, log S, and log V.P.) of the pesticides (EU Pesticides database) and MS/MS and μECD/NPD analyte responses were found applying a PCA.

**Fig. 3:**
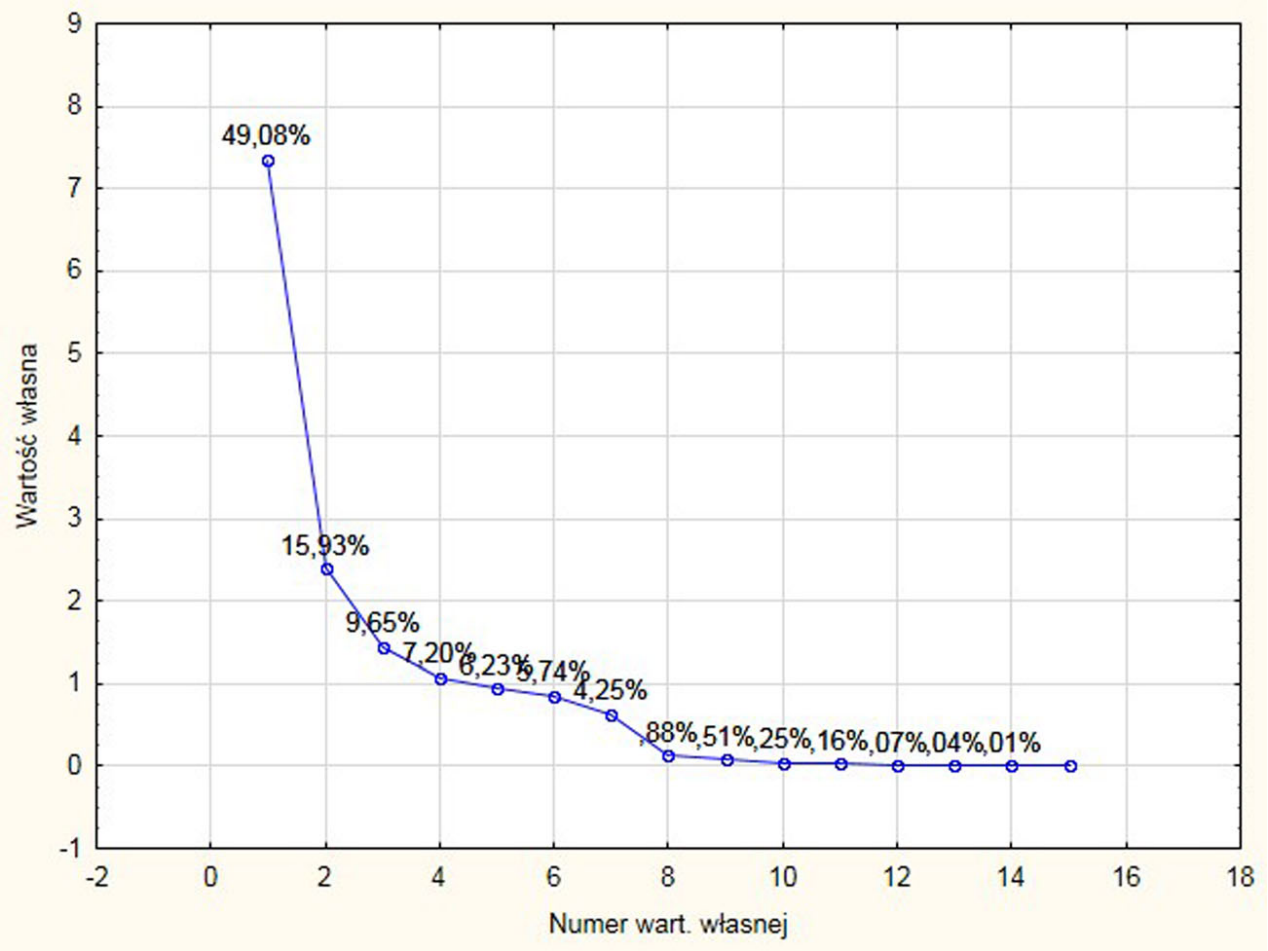
Scree plot graph presenting the eigenvalue against the component number

The first four principal components summarized about 81.86% of the available information (the loadings associated with principal components with eigenvalues larger than 1 were as follows: PC1 49.08%, PC2 15.93%, PC3 9.65%, and PC4 7.20%). PC3 and PC4 eigenvalues were relatively small. The first PC1 and the second principal component PC2 described more 65% of the variation and were further analyzed according to scree plot showing “elbow” on graph after PC2 (Fig. 3).

**Fig. 4:**
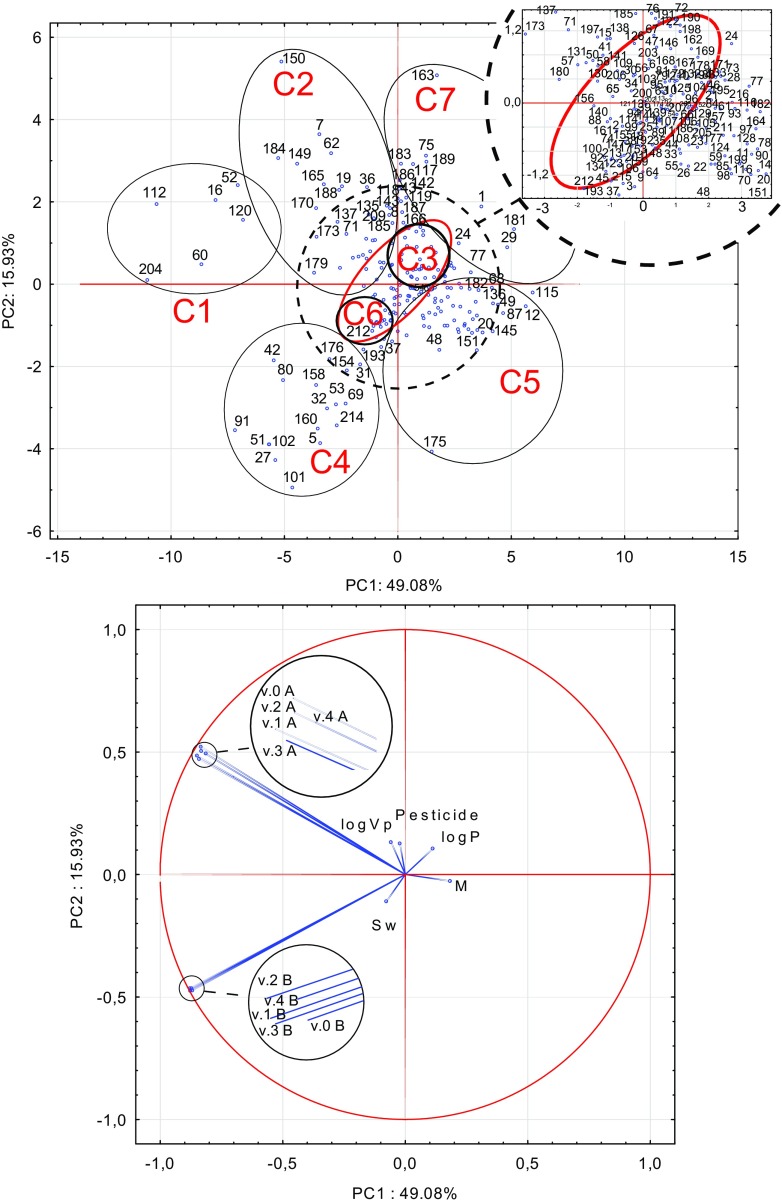
Score and loading plot of the first (PC1) vs. second principal component (PC2)

Figure 4 presents score and loading plot of the first (PC1) vs. second principal component (PC2). The compounds having the highest PC1 scores were bifenazat (ID 12; 2.01%), bromacyl (16; 4.08%), dicloran (51, 2.02%), dicofol (52, 3.14%), dimoxystrobin (60, 4.72%), flufenacet (91; 3.25%), fuberidazol (102; 2.03%), imazalil (112; 7.15%), iprodione (115; 2.23%), isoprocarb (120; 2.93%), and thiabendazole (204; 7.69%).

The correlations between the matrix effect of 216 pesticides and their physicochemical parameters were found and the groups of pesticides with similar properties were defined dividing them into seven clusters (Fig. 4). The ID number of each pesticide is given in Supplementary Table S2. Interpreting the scores and loadings, the pesticides were categorized into cluster: (C1) with high matrix effect value determined by GC/MS/MS (about 150–160%) (e.g., 16, 52, 60, 112, 120, 204); (C2) highly polar with negative log*P* (e.g., 7, 8, 15, 19, 30, 56, 58, 62, 65, 71, 127, 130, 131, 137, 143, 149, 150, 165, 170, 180, 184, 188, 206, 209 with exception 34, 36, 50, 57, 109, 135, 138, 141, 185, 126, 173, 179, 180 with low value of log*P* < 1.3); (C3) with log*P* > 3 (e.g., 21, 24, 28, 63, 73, 79, 81, 83, 95, 103, 132, 162, 168, 169, 172, 178, 182, 190, 194, 195, 198, 200, 202, 216, 210, except 10, 133, 171); (C4) with high matrix effect value determined by GC/EC/NP (e.g., 27, 42, 51, 80, 91, 101, 102 with ME = 180–190%; 5, 31, 32, 53, 69, 158, 160, 214, 212 with ME = 130–160% and 37, 45, 154, 176, 193 with ME = 110–130%); (C5) with molecular mass >300 g/mol (e.g., 11, 12, 14, 20, 22, 49, 59, 68, 77, 78, 85, 87, 98, 115, 116, 136, 145, 175 except 48, 90 with M below 300 g/mol); (C6) very soluble (e.g., 2, 9, 18, 74, 88, 92, 100, 121, 123, 134, 140, 147, 159, 215 except 3, 193) and (C7) with ME 50–90% on GC/EC/NP (e.g., 1, 24, 29, 75, 117, 163, 181, 183, 186, 189).

The dominant variables influencing the matrix effect were polarity and solubility of pesticides concentrating the largest number of compounds. Thus, red cluster including C3 + C6 and other compounds were separated consisting of highly soluble (Sw > 4 mg/l) and nonpolar (log*P* > 3) pesticides.

In summary, both the matrix effect and recovery depended on applied detection system. Additionally, gas chromatography with selective detectors offers only limited specificity and does not provide unambiguous identification. Therefore, tandem mass spectrometry in conjunction with gas chromatography is a very powerful combination for identification of analytes in the soil extract. The selection of three transitions, one for quantification and two for confirmation, gives excellent selectivity and sensitivity and the possibility of safe identification (Table S2).

### Quality control procedure

Certified Reference Material (CRM, ERA—A Water Company) was used to verify accuracy of the proposed procedure for the quantitative determination of variety range of pesticides in soils. Certified values of CRM with uncertainties were compared with the values obtained from the analysis of soil samples using QuEChERS method without cleanup analyzing by GC/MS/MS and GC-μECD/NPD (Table 1).

**Table 1:** Results of analyzed pesticides by QuEChERS without cleanup method using the gas chromatography with MS/MS and EC/NP detection and value of Certified Reference Material

Active substance	Certified value (mg/kg) ± uncertainty (%)	Acceptance limits (mg/kg)	Laboratory results ± U (mg/kg)	
			μECD/NPD	MS/MS
Carbaryl	0.870 ± 0.652	0.416–0.957	0.653 ± 0.1241	0.726 ± 0.1116
Carbofuran	2.060 ± 0.668	0.793–2.390	1.012 ± 0.2948	1.452 ± 0.2755
Propham	0.886 ± 5.620	0.211–1.220	0.438 ± 0.0919	0.668 ± 0.1002

The results for carbaryl, carbofuran, and propham obtained in two systems of detection were very comparable to the assigned true concentrations, within the interlaboratory uncertainty intervals. Overall, the results were in acceptance value; moreover, the GC/MS/MS results are a bit higher than the GC-μECD/NPD, in correspondence with reported respective lower recoveries (70, 73, and 67% for GC-μECD/NPD and 75, 79, 84% for GC/MS/MS).

### Application to real sample

The results of the method were applied to 263 soil samples from the north-eastern Poland collected in 2015 are in Table 2. Of the samples, 58.2% (153) were found pesticide residues. Pesticides like organochlorines banded in Europe as plant protection products were detected in soil samples, due to their persistence in the environment. P,p’ DDT (23.5% of positive samples) and p,p’ DDE (17% of positive samples) were the most frequently detected. The highest concentration was found for pendimethalin (1.63 mg/kg). The recovery factors were used for calculating pesticide concentration only in the case of pesticides that indicate recoveries outside the range 70*–*120% (within the range 60*−*69% and 121*−*130%) (SANCO 2013).

**Table 2:** Pesticide residues found in soil real samples (total 263 samples)

Pesticide (category)	No. and frequency of positive samples (%)	LOQ^a^ (mg/kg)	Concentration range (mg/kg)	
			Min	Max
Acetochlor (H)	2 (0.3)	0.005	0.04	0.12
Azoxystrobin (F)	1 (0.2)	0.005	0.03	
Boscalid (F)	2 (0.3)	0.005	0.07	0.12
Bupirimate (F)	2 (0.3)	0.005	0.01	0.03
Chlorpyrifos (I)	14 (2.4)	0.005	0.01	0.27
Cypermethrin (I)	2 (0.3)	0.005	0.01	
Cyprodinil (F)	2 (0.3)	0.005	0.01	0.08
p,p’ DDD (I)	16 (2.7)	0.005	0.003	0.037
p,p DDE (I)	101 (17.0)	0.005	0.003	0.055
o,p’ DDT (I)	7 (1.2)	0.005	0.003	0.042
p,p’ DDT (I)	140 (23.5)	0.005	0.003	0.265
Epoxiconazole (F)	4 (0.7)	0.005	0.02	
Fenazaquin (A)	1 (0.2)	0.005	0.03	
Fludioxonil (F)	1 (0.2)	0.005	0.03	
Lenacil (H)	11 (1.9)	0.005	0.02	0.75
Methoxychlor (DMDT) (I)	2 (0.3)	0.005	0.01	
Napropamide (H)	5 (0.8)	0.005	0.03	0.06
Oxyflurofen (H)	1 (0.2)	0.005	0.14	
Pendimethalin (H)	19 (3.2)	0.005	0.01	1.63
Spirodiclofen (A)	3 (0.5)	0.01	0.02	
Simazine (H)	3 (0.5)	0.005	0.01	0.03
Tebuconazole (F)	1 (0.2)	0.005	0.02	
Tetraconazole (F)	6 (1.0)	0.005	0.01	

*H* herbicide, *F* fungicide, *I* insecticide, *A* acaricide

^a^Limit of quantification (GC/MS/MS)

**Fig. 5:**
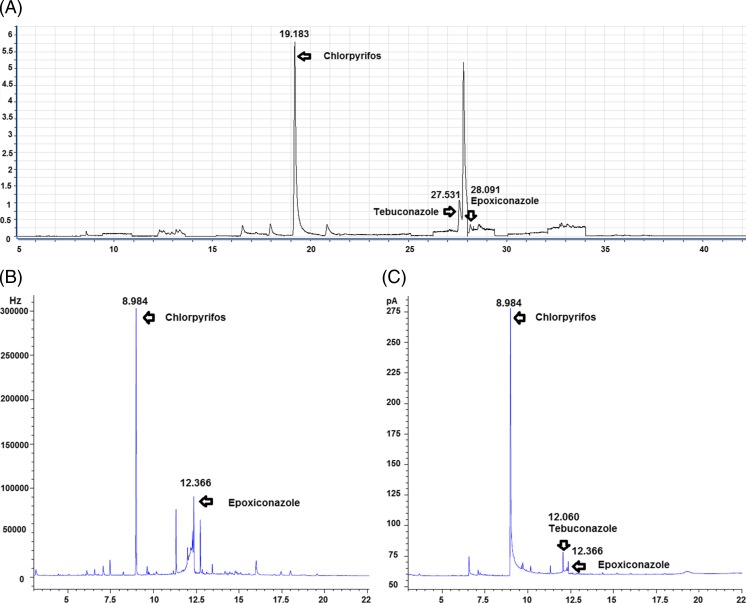
Chromatogram of real soil sample containing chlorpyrifos (0.06 mg/kg), epoxiconazole (0.06 mg/kg), and tebuconazole (0.24 mg/kg) using **a** GC/MS/MS, **b** GC-μECD, and **c** GC-NPD

Typical chromatograms of real sample extract that contain three pesticide residues chlorpyrifos, epoxiconazole, and tebuconazole using GC/MS/MS and GC-μECD/NPD are shown Fig. 5.

Therefore, the objective of this study is relevant to monitoring research of pesticide residues by innovative and convenience of QuEChERS method for the determination of over 210 compounds.

## Conclusions

The influence of modifications of QuEChERS on recovery and matrix effect during the multi-residue analysis of wide range of pesticides in soil was compared.

For the first time for sensitive identification and determination, a broad scope of pesticides and metabolites (216) in soil samples using gas chromatography (GC) coupled with tandem mass spectrometry (MS/MS) and dual system electron capture/phosphorous-nitrogen detectors (μECD/NPD). It was a very challenging task, because soil is a complex matrix and compounds are characterized by great structural variability and physicochemical properties, which cause many analytical problems.

The optimal validation parameters for procedure without cleanup step were obtained and this modification allowed for gently reducing the time of analysis. Nevertheless, validation parameters for gas chromatography coupled with mass spectrometry fulfilled the criteria of pesticide residue guide largely than selective system of detection μECD/NPD for several pesticides. The analysis covered a wide range of pesticides and may be used single and complementary.

For the QuEChERS method without cleanup, recoveries for 216 pesticides and metabolites were satisfactory; they ranged 65–116% (RSD ≤17%) and 60–112% (RSD ≤18%) for MS/MS and μEC/NP, respectively. GC/MS/MS gave smaller matrix effects showing suppression or enhancement in the range (−25 to 74%), contrary to μEC/NP (−45 to 96%).

For better understanding on matrix effect PCA analysis, a powerful statistical tool was used. The correlations between the selected physicochemical properties of 216 pesticides and metabolites were found and the key parameters influencing the matrix effect were polarity and solubility of pesticides.

Compared to other works involving pesticide residue analysis in soil, the proposed QuEChERS method has considerable superiorities in respect of target number, sample extraction procedure, and method validation.

In conclusion, the proposed method meets the EU criteria and MRL levels and is thus useful for routine residue analysis of pesticides in soil matrices and detecting p,p’ DDT, p,p’ DDE, pendimethalin, p,p’ DDD, chlorpyrifos, lenacil, and other.

The applicability of QuEChERS for this type of organic contaminants as well as the excellent sensitivity obtained using GC/MS/MS/EC/NP has been demonstrated.

## Electronic supplementary material

Table S1 Acquisition and chromatographic parameters for GC/MS/MS analysis of the 216 pesticides and Internal Standard (IS). (DOCX 30 kb.)

Table S2 Matrix effects, linear range, correlation coefficients, recoveries, RSDs and expanded uncertainties (U) of 216 pesticides QuEChERS method without cleanup step analysis by GC-μECD-NPD and GC/MS/MS. (DOCX 73 kb.)

## References

[CR1] Anastassiades M, Lehotay SJ, Stajnbaher D, Schenck FJ (2003). Residues and trace elements fast and easy multiresidue method employing acetonitrile extraction partitioning and “dispersive solid-phase extraction”. J AOAC Int.

[CR2] Anastassiades M, Maštovská K, Lehotay SJ (2013). Evaluation of analyte protectants to improve gas chromatographic analysis of pesticides. J Chromatogr A.

[CR3] Arias JLO, Rombaldi C, Caldas SS, Primel EG (2014). Alternative sorbents for the dispersive solid-phase extraction step inquick, easy, cheap, effective, rugged and safe method for extraction of pesticides from rice paddy soils with determination by liquid chromatography tandem mass spectrometry. J Chromatogr A.

[CR4] Asensio-Ramos M, Hernandez-Borges J, Ravelo-Perez LM, Rodriguez-Delgado MA (2010). Evaluation of a modified QuEChERS method for the extraction of pesticides from agricultural, ornamental and forestal soils. Anal Bioanal Chem.

[CR5] Bargańska Ż, Ślebioda M, Namieśnik J (2013). Pesticide residues levels in honey from apiaries located of Northern Poland. Food Control.

[CR6] Bruzzoniti MC, Checchini L, De Carlo RM, Orlandini S, Rivoira L, Del Bubba M (2014). QuEChERS sample preparation for the determination of pesticides and other organic residues in environmental matrices: a critical review. Anal Bioanal Chem.

[CR7] Cajka T, Sandy C, Bachanova V, Drabova L, Kalachova K, Pulkrabova J, Hajslova J (2012). Streamlining sample preparation and gas chromatography-tandem mass spectrometry analysis of multiple pesticide residues in tea. Anal Chim Acta.

[CR8] Caldas SS, Bolzan CM, Cerqueira MB, Tomasini D, Furlong EB, Fagundes C, Primel EG (2011). Evaluation of a modified QuEChERS extraction of multiple classes of pesticides from a rice paddy soil by LC-APCI-MS/MS. J Agric Food Chem.

[CR9] Chen L, Li XS, Wang ZQ, Pan CP, Jin RC (2010). Residue dynamics of procymidone in leeks and soil in greenhouses by smoke generator application. Ecotox Environ Safe.

[CR10] Correia-Sá L, Fernandes VC, Carvalho M, Calhau C, Domingues VF, Delerue-Matos C (2012). Optimization of QuEChERS method for the analysis of organochlorine pesticides in soils with diverse organic matter. J Sep Sci.

[CR11] Dąbrowska H, Dąbrowski L, Biziuk M, Gaca J, Namieśnik J (2003). Solid-phase extraction clean-up of soil and sediment extracts for the determination of various types of pollutants in a single run. J Chromatogr A.

[CR12] Durović R, Dordević T, Radivojević L, Šantrić L, Gajić Umiljendić J (2012). Multiresidue analysis of pesticides in soil by liquid-solid extraction procedure. Pestic Phytomed.

[CR13] Erney DR, Pawlowski TM, Poole CF (1997). Matrix-induced peak enhancement of pesticides in gas chromatography: is there a solution?. J High Resol Chromatogr.

[CR14] EU Pesticides database. Available at http://ec.europa.eu/sanco_pesticides/public/index.cfm

[CR15] Fernandes VC, Domingues VF, Mateus N, Delerue-Matos C (2013). Multiresidue pesticides analysis in soils using modified QuEChERS with disposable pipette extraction and dispersive solid-phase extraction. J Sep Sci.

[CR16] Fuentes E, Báez ME, Labra R (2007). Parameters affecting microwave-assisted extraction of organophosphorus pesticides from agricultural soil. J Chromatogr A.

[CR17] Guo L, Lee HK (2013). Microwave assisted extraction combined with solvent bar microextraction for one-step solvent-minimized extraction, cleanup and preconcentration of polycyclic aromatic hydrocarbons in soil samples. J Chromatogr A.

[CR18] Harrison R, Bull I, Michaelides K (2013). A method for the simultaneous extraction of seven pesticides from soil and sediment. Anal Methods.

[CR19] He Z, Wang L, Peng Y, Luo M, Wang W, Liu X (2015). Multiresidue analysis of over 200 pesticides in cereals using a QuEChERS and gas chromatography-tandem mass spectrometry-based method. Food Chem.

[CR20] Kaczyński P, Łozowicka B, Jankowska M, Hrynko I (2016). Rapid determination of acid herbicides in soil by liquid chromatography with tandem mass spectrometric detection based on dispersive solid phase extraction. Talanta.

[CR21] Kinsella B, O’Mahony J, Malone E, Moloney M, Cantwell H, Furey A, Danaher M (2009). Current trends in sample preparation for growth promoter and veterinary drug residue analysis. J Chromatogr A.

[CR22] Kolberg DI, Prestes OD, Adaime MB, Zanella R (2011). Development of a fast multiresidue method for the determination of pesticides in dry samples (wheat grains, flour and bran) using QuEChERS based method and GC-MS. Food Chem.

[CR23] Kruve A, Künnapas A, Herodes K, Leito I (2008). Matrix effects in pesticide multi-residue analysis by liquid chromatography-mass spectrometry. J Chromatogr A.

[CR24] Lesueur C, Gartner M, Mentler A, Fuerhacker M (2008). Comparison of four extraction methods for the analysis of 24 pesticides in soil samples with gas chromatography-mass spectrometry and liquid chromatography-ion trap-mass spectrometry. Talanta.

[CR25] Lehotay SJ, Son KA, Kwon H, Koesukwiwat U, Fu W, Mastovska K, Hoh E, Leepipatpiboon N (2010). Comparison of QuEChERS sample preparation methods for the analysis of pesticide residues in fruits and vegetables. J Chromatogr A.

[CR26] Li Y, Dong F, Liu X, Xu J, Li J, Kong Z, Chen X, Liang X, Zheng Y (2012). Simultaneous enantioselective determination of triazole fungicides in soil and water by chiral liquid chromatography/tandem mass spectrometry. J Chromatogr A.

[CR27] Lozano A, Rajski Ł, Belmonte-Valles N, Ucles A, Ucles S, Mezcua M, Fernandez-Alba AR (2012). Pesticide analysis in teas and chamomile by liquid chromatography and gas chromatography tandem mass spectrometry using a modified QuEChERS method: validation and pilot survey in real samples. J Chromatogr A.

[CR28] Łozowicka B, Jankowska M, Rutkowska E, Kaczyński P, Hrynko I (2012). Comparison of extraction techniques by matrix solid phase dispersion and liquid–liquid for screening 150 pesticides from soil, and determination by gas chromatography. Pol J Environ Stud.

[CR29] Łozowicka B, Rutkowska E, Hrynko I (2015). Simultaneous determination of 223 pesticides in tobacco by GC with simultaneous electron capture and nitrogen-phosphorous detection and mass spectrometric confirmation. Open Chem.

[CR30] Łozowicka B, Ilyasova G, Kaczynski P, Jankowska M, Rutkowska E, Hrynko I, Mojsak P, Szabunko J (2016). Multi-residue methods for the determination of over four hundred pesticides in solid and liquid high sucrose content matrices by tandem mass spectrometry coupled with gas and liquid chromatograph. Talanta.

[CR31] Mantzos N, Karakitsou A, Zioris I, Leneti E, Konstantinou I (2013). QuEChERS and solid phase extraction methods for the determination of energy crop pesticides in soil, plant and runoff water matrices. Inter J Environ Anal Chem.

[CR32] Martinez Vidal JL, Padilla Sanchez JA, Plaza-Bolanos P, Garrido Frenich A, Romero-Gonzalez R (2010). Use of pressurized liquid extraction for the simultaneous analysis of 28 polar and 94 non-polar pesticides in agricultural soils by GC/QqQ-MS/MS and UPLC/QqQ-MS/MS. J AOAC Int.

[CR33] Masiá A, Vásquez K, Campo J, Picó Y (2015). Assessment of two extraction methods to determine pesticides in soils, sediments and sludges. Application to the Túria River Basin J Chromatogr A.

[CR34] Mol HGJ, Rooseboom A, van Dam R, Roding M, Arondeus K, Sunarto S (2007). Modification and re-validation of the ethyl acetate-based multi-residue method for pesticides in produce. Anal Bioanal Chem.

[CR35] Moreno D, Ferrera VZ, Rodriguez J (2006). Sample extraction method combining micellar extraction-SPME and HPLC for the determination of organochlorine pesticides in agricultural soils. J Agr Food Chem.

[CR36] Moreno López A, Moreno López L, Pineda Lucas JL, Stevens J (2014) Multiresidue analysis of pesticides in olive samples using GC/MS/MS. application note, food testing and agriculture. Agilent Technologies:1–8

[CR37] Naeeni MH, Yamini Y, Rezaee M (2011). Combination of supercritical fluid extraction with dispersive liquid-liquid microextraction for extraction of organophosphorus pesticides from soil and marine sediment samples. Journal Supercrit Fluids.

[CR38] Pastor-Belda M, Garridob I, Campilloa N, Vinasa P, Hellínb P, Floresb P, Fenollb J (2015). Dispersive liquid-liquid microextraction for the determination of new generation pesticides in soils by liquid chromatography and tandem mass spectrometry. J Chromatogr A.

[CR39] Pinto CG, Martin SH, Pavon JLP, Cordero BM (2011). A simplified Quick, Easy, Cheap, Effective, Rugged and Safe approach for the determination of trihalomethanes and benzene, toluene, ethylbenzene and xylenes in soil matrices by fast gas chromatography with mass spectrometry detection. Anal Chim Acta.

[CR40] Rashid A, Nawaz S, Barker H, Ahmad I, Ashraf M (2010). Development of a simple extraction and clean-up procedure for determination of organochlorine pesticides in soil using gas chromatography-tandem mass spectrometry. J Chromatogr A.

[CR41] Rouvière F, Buleté A, Cren-Olivé C, Arnaudguilhem C (2012). Multiresidue analysis of aromatic organochlorines in soil by gas chromatography-mass spectrometry and QuEChERS extraction based on water/dichloromethane partitioning. Comparison with accelerated solvent extraction. Talanta.

[CR42] SANCO (2013) Document No. SANCO/12571/2013. Guidance document on analytical quality control and validation procedures for pesticide residues in food and feed

[CR43] Sanghi R, Kannamkumarath SS (2004). Comparison of extraction methods by soxhlet, sonicator, and microwave in the screening of pesticide residues from solid matrices. J Anal Chem.

[CR44] Tor A, Aydin ME, Ozcan S (2006). Ultrasonic solvent extraction of organochlorine pesticides from soil. Anal Chim Acta.

[CR45] Varga R, Somogyvári I, Eke Z, Torkos K (2011). Determination of antihypertensive and anti-ulcer agents from surface water with solid-phase extraction–liquid chromatography–electrospray ionization tandem mass spectrometry. Talanta.

[CR46] Vera J, Correia-Sá L, Paíga P, Bragança I, Fernandes VC, Domingues VF, Delerue-Matos C (2013). QuEChERS and soil analysis. An overview. Sample Perp.

[CR47] Wang YH, Du LW, Zhou XM, Tan HH, Bai LY, Zeng DQ, Tian H (2012). QuEChERS extraction for high performance liquid chromatographic determination of pyrazosulfuron-ethyl in soils. J Chem Soc Pak.

[CR48] Wu M, Hu J (2014). Residue analysis of fosthiazate in cucumber and soil by QuEChERS and GC-MS. Chem Pap.

[CR49] Zhang K, Wong JW, Yang P, Tech K, Dibenedetto AL, Lee NS (2011). Multiresidue pesticide analysis of agricultural commodities using acetonitrile salt-out extraction, dispersive solid-phase sample clean-up, and high-performance liquid chromatography-tandem mass spectrometry. J Agr Food Chem.

