# Supplementary material for: Influence of QuEChERS modifications on recovery and matrix effect during the multi-residue pesticide analysis in soil by GC/MS/MS and GC/ECD/NPD
Source: Environ Sci Pollut Res Int. 2017 Jan 16;24(8):7124–38. doi: 10.1007/s11356-016-8334-1 (PMC5383684; doi:10.1007/s11356-016-8334-1)
Supplement: Supplementary file 1 — Acquisition and chromatographic parameters for GC/MS/MS analysis of the 216 pesticides and Internal Standard (IS). (DOCX 30 kb.) [file 11356_2016_8334_MOESM1_ESM.docx]

**Table S1.** Acquisition and chromatographic parameters for GC-MS/MS analysis of the 216 pesticides and Internal Standard (IS).

| No. | Active substance  (pesticide type) | *t_R_* min | MRM transitions m/z (collision energy eV) | | |
| --- | --- | --- | --- | --- | --- |
|  |  |  | Quantitative ion pairs | Qualitative ion pairs I | Qualitative ion pairs II |
|  | Methamidophos (I) | 5.603 | 141 > 95 (5) | 95 > 79 (10) | 95 > 64 (10) |
|  | Dichlorvos (I) | 5.784 | 109 > 79 (5) | 185 > 93 (10) | 145 > 109 (10) |
|  | Dichlobenil (H) | 6.701 | 171 > 100 (25) | 171 > 136 (15) | 173 > 100 (25) |
|  | Mevinphos (I) | 7.534 | 127 > 109 (10) | 127 > 95 (15) | 192 > 127 (10) |
|  | Propham (H) | 7.855 | 119 > 91 (10) | 137 > 93 (10) | 179 > 93 (15) |
|  | Methacrifos (F) | 8.497 | 208 > 180 (5) | 125 > 47 (10) | 125 > 79 (5) |
|  | Oxamyl (I) | 8.986 | 98 > 58 (10) | 98 > 69 (10) | 162 > 115 (10) |
|  | Isoprocarb (I) | 9.031 | 121 > 77(20) | 136 > 121 (10) | 121 > 103 (10) |
|  | Heptenophos (I) | 9.661 | 124 > 89 (10) | 124 > 63 (35) | 109 > 79 (5) |
|  | DEET (I) | 9.672 | 119 > 91 (10) | 119 > 65 (20) | 91 > 65 (10) |
|  | Tecnazene (F) | 10.153 | 261 > 203 (20) | 215 > 179 (5) | 259 > 201 (5) |
|  | Flonicamid (I) | 10.190 | 174 > 146 (10) | 174 > 126 (30) | 146 > 69 (30) |
|  | Propoxur (I) | 10.256 | 110 > 63 (25) | 110 > 64 (15) | 152 > 110 (10) |
|  | Propachlor (H) | 10.265 | 120 > 77 (10) | 176 > 57 (10) | 120 > 92 (10) |
|  | Difenyloamina (F) | 10.418 | 169 > 168 (15) | 168 > 167 (15) | 167 > 166 (20) |
|  | Ethoprophos (I) | 10.661 | 158 > 97 (15) | 158 > 114 (5) | 139 > 97 (5) |
|  | Chlorpropham (H) | 10.977 | 153 > 125 (10) | 153 > 90 (25) | 127 > 65 (25) |
|  | Dicrotophos (I) | 11.394 | 127 > 109 (15) | 127 > 95 (15) | 193 > 127 (5) |
|  | Pencycuron (F) | 11.542 | 208. >125 (20) | 180 > 125 (20) | 180 > 89 (30) |
|  | Trifluralin (H) | 11.557 | 306 > 264 (5) | 264 > 160 (15) | 264 > 206 (5) |
|  | Cadusafos (I) | 11.710 | 159 > 97 (15) | 159 > 131 (5) | 158 > 97 (15) |
|  | Phorate (I) | 11.851 | 121 > 65 (10) | 121 > 47 (30) | 129 > 65 (15) |
|  | HCH alpha (I) | 11.970 | 217 > 181 (5) | 219 > 183 (5) | 181 > 145 (15) |
|  | HCB (I) | 12.261 | 284 > 214 (30) | 284 > 249 (15) | 289 > 212 (30) |
|  | Desmedipham (H) | 12.410 | 135 > 79 (15) | 135 > 52 (25) | 109 > 80 (15) |
|  | Dicloran (F) | 12.461 | 206 > 176 (10) | 160 > 124 (10) | 124 > 73 (10) |
|  | Dimethoate (I) | 12.563 | 86 > 46 (15) | 93 > 63 (10) | 87 > 86 (5) |
|  | Simazine (H) | 12.809 | 201 > 173 (5) | 173 > 172 (5) | 173 > 138 (5) |
|  | Carbofuran (I) | 12.925 | 164 > 149 (10) | 149 > 121 (5) | 149 > 77 (30) |
|  | Atrazine (H) | 13.065 | 215 > 58 (10) | 215 > 200 (5) | 200 > 122 (5) |
|  | Clomazone (H) | 13.128 | 125 > 89 (15) | 204 > 107 (20) | 125 > 99 (15) |
|  | HCH beta (I) | 13.150 | 181 > 145 (15) | 217 > 181 (5) | 219 > 183 (5) |
|  | Propazine (H) | 13.275 | 214 > 172 (10) | 229 > 58 (10) | 229 > 214 (5) |
|  | HCH gamma (lindane) (I) | 13.337 | 217 > 181 (5) | 181 > 145 (15) | 219 > 183(5) |
|  | Terbuthylazine (H) | 13.705 | 173 > 172 (5) | 229 > 173 (5) | 173 > 138 (5) |
|  | Propyzamide (H) | 13.855 | 173 > 145 (15) | 175 > 147 (15) | 173 > 109 (30) |
|  | Pyrimethanil (F) | 14.039 | 198 > 183 (15) | 198 > 118 (35) | 198 > 158 (20) |
|  | Diazinon (I) | 14.369 | 137 > 84 (10) | 137 > 54 (20) | 199 > 93 (15) |
|  | Paraoxon methyl (I) | 14.460 | 109 > 79 (5) | 230 > 106 (15) | 230 > 136 (5) |
|  | Chlorothalonil (F) | 14.697 | 264 > 168 (25) | 264 > 229 (20) | 266 > 231 (20) |
|  | Tefluthrin (I) | 14.977 | 177 > 127 (15) | 197 > 141 (10) | 177 > 87 (30) |
|  | Formothion (I) | 15.427 | 170 > 93 (5) | 125 > 47 (15) | 125 > 79 (5) |
|  | Metobromuron (H) | 15.499 | 197 > 90 (20) | 199 > 90 (20) | 199 > 171 (10) |
|  | Pirimicarb (I) | 15.580 | 238 > 166 (10) | 166 > 55 (20) | 166 > 96 (15) |
|  | Cyprazine (H) | 16.104 | 212 > 170 (10) | 212 > 109 (25) | 227 > 212 (10) |
|  | Dimethenamid (H) | 16.108 | 230 > 154 (10) | 154 > 111 (10) | 232 > 154 (10) |
|  | Metribuzin (H) | 16.159 | 198 > 82 (15) | 198 > 55 (30) | 144 > 128 (10 |
|  | Acetochlor (H) | 16.434 | 174 > 146 (10) | 146 > 131 (10) | 223 > 132 (20) |
|  | Fuberidazole (F) | 16.486 | 184 > 156 (10) | 184 > 155 (30) | 183 > 155 (10) |
|  | Parathion methyl (I) | 16.487 | 269 > 109 (10) | 125 > 47 (10) | 125 > 79 (5) |
|  | Chlorpyrifos methyl (I) | 16.488 | 125 > 47 (15) | 125 > 79 (5) | 286 > 93 (20) |
|  | Vinclozolin (F) | 16.529 | 125 > 47 (15) | 125 > 79 (5) | 286 > 93 (20) |
|  | Heptachlor (I) | 16.662 | 100 > 58 (10) | 100 > 72 (5) | 198 > 126 (5) |
|  | Tolclofos methyl (F) | 16.693 | 265 > 250 (15) | 265 > 93 (25) | 125 > 47 (15) |
|  | Carbaryl (I) | 16.708 | 144 > 115 (20) | 144 > 116 (10) | 116 > 115 (10) |
|  | Malaoxon (I) | 16.778 | 127 > 99 (5) | 127 > 55 (5) | 99 > 71 (5) |
|  | Fenchlorfos (I) | 17.217 | 285 > 270 (15) | 288 > 272 (15) | 125 > 47 (15) |
|  | Metalaxyl (F) | 17.235 | 220 > 192 (5) | 234 > 146 (20) | 234 > 174 (10) |
|  | Paraoxon ethyl (I) | 17.242 | 109 > 81 (10) | 109 > 91 (5) | 149 > 119 (5) |
|  | Prometrine (H) | 17.253 | 226 > 184 (10) | 199 > 184 (5) | 241 > 184 (10) |
|  | Terbutryn (H) | 17.882 | 185 > 170 (5) | 241 > 170 (15) | 185 > 111 (15) |
|  | Fenitrothion (I) | 17.967 | 125 > 47 (15) | 125 > 79 (5) | 277 > 260 (5) |
|  | Quinoclamine (H) | 18.050 | 207 > 172 (10) | 209 > 172 (10) | 172 > 89 (20) |
|  | Pirimiphos methyl (I) | 18.203 | 290 > 125 (20) | 233 > 151 (5) | 233 > 125 (5) |
|  | Bromacyl (H) | 18.245 | 205 > 188 (15) | 207 > 190 (15) | 205 > 162 (15) |
|  | Dichlofluanid (F) | 18.288 | 123 > 77 (20) | 224 > 123 (10) | 226 > 123 (10) |
|  | Aldrine (I) | 18.391 | 263 > 193 (35) | 255 > 220 (20) | 263 > 191 (35) |
|  | Malathion (I) | 18.701 | 127 > 99 (5) | 173 > 99 (15) | 158 > 125 (5) |
|  | Metolachlor (H) | 18.809 | 238 > 162 (10) | 162 > 133 (15) | 240 > 162 (10) |
|  | Fention (I) | 19.013 | 278 > 109 (15) | 125 > 47 (10) | 124.9 > 79 (5) |
|  | Dicofol (A) | 19.099 | 250 > 139 (20) | 139 > 111 (20) | 139 > 75 (20) |
|  | Chlorpyrifos (I) | 19.133 | 197 > 169 (15) | 199 > 171 (15) | 314 > 258 (15) |
|  | Fenpropimorph (F) | 19.157 | 128 > 70 (10) | 128 > 110 (5) | 128 > 86 (10) |
|  | Parathion ethyl (I) | 19.165 | 139 > 109 (5) | 291 > 109 (10) | 139 > 81 (15) |
|  | Cyanazine (H) | 19.290 | 212 > 123 (15) | 212 > 151 (15) | 198 > 91 (10) |
|  | Triadimefon (F) | 19.303 | 208 > 181 (5) | 208 > 111 (20) | 128 > 65 (20) |
|  | Isocarbophos (A) | 19.503 | 120 > 92 (10) | 136 > 108 (15) | 121 > 65 (15) |
|  | Flufenacet (H) | 19.555 | 151 > 136 (10) | 151 > 95 (30) | 123 > 95 (20) |
|  | Tetraconazole (F) | 19.769 | 171 > 136 (10) | 336 > 218 (20) | 336 > 204 (30) |
|  | Flurochloridone (H) | 19.874 | 187 > 159 (10) | 311 > 174 (15) | 145 > 95 (15) |
|  | Bromofos methyl (I) | 19.964 | 125 > 47 (10) | 331 > 316 (15) | 125 > 79 (5) |
|  | Thiametoxam (I) | 19.996 | 247 > 182 (10) | 211 > 139 (10) | 212 > 125 (10) |
|  | Fostiazate (I) | 20.124 | 195 > 103 (5) | 195 > 60 (20) | 199 > 102 (5) |
|  | Cyprodinil (F) | 20.468 | 225 > 224 (10) | 224 > 208 (20) | 226 > 225 (10) |
|  | Pirimiphos ethyl (I) | 20.578 | 318 > 166 (10) | 318 > 182 (10) | 152 > 84 (10) |
|  | Isofenfos metylowy (I) | 20.620 | 199 > 121 (10) | 199 > 167 (10) | 167 > 123 (5) |
|  | Metazachlor (H) | 20.710 | 133 > 132 (10) | 132 > 117 (15) | 209 > 132 (15) |
|  | Heptachlor epoxide (I) | 20.846 | 183 > 155 (15) | 183 > 119 (25) | 217 > 182 (20) |
|  | Pendimethalin (H) | 20.893 | 252>162.2(10) | 252 > 161 (15) | 162 > 161 (10) |
|  | Thiabendazole (I) | 20.917 | 201 > 174 (15) | 202 > 175 (15) | 174 > 65 (30) |
|  | Penconazole (F) | 20.923 | 248 > 192 (15) | 248 > 157 (25) | 159 > 89 (35) |
|  | Captan (F) | 21.123 | 151 > 80 (5) | 149 > 79 (10) | 151 > 79 (15) |
|  | Tolylfluanid (F) | 21.128 | 137 > 91 (20) | 238 > 137 (15) | 137 > 65 (30) |
|  | Chlorfenvinphos (I) | 21.483 | 267 > 159 (15) | 323 > 267 (10) | 269 > 161 (15) |
|  | Folpet (F) | 21.491 | 260 > 130 (15) | 262 > 130 (15) | 260 > 232 (5) |
|  | Isofenphos (I) | 21.515 | 213 > 121 (10) | 213 > 185 (5) | 185 > 121 (5) |
|  | Quinalphos (I) | 21.551 | 146 > 118 (10) | 146 > 91 (30) | 157 > 129 (15) |
|  | Triadimenol (F) | 21.598, 22.003 | 128 > 65 (25) | 168 > 70 (10) | 128 > 100 (10) |
|  | Phentoate (I) | 21.619 | 274 > 121 (10) | 274 > 125 (15) | 121 > 77 (25) |
|  | Mecarbam (I) | 21.637 | 159 > 131 (5) | 131 > 74 (5) | 131 > 86 (10) |
|  | Fipronil (I) | 21.770 | 351 > 255 (15) | 367 > 213 (25) | 255 > 228 (15) |
|  | Procymidone (F) | 21.863 | 96 > 67 (10) | 96 > 53 (15) | 283 > 96 (10) |
|  | Hexythiazox (A) | 22.044 | 227 > 149 (10) | 184 > 149 (10) | 184 > 115 (20) |
|  | Methidathion (I) | 22.204 | 145 > 85 (5) | 145 > 58 (15) | 85 > 58 (5) |
|  | Triflumizole (F) | 22.246 | 206 > 179 (15) | 206 > 186 (10) | 179 > 144 (15) |
|  | Bromofos ethyl (I) | 22.443 | 359 > 303 (15) | 303 > 285 (15) | 242 > 97 (30) |
|  | Paclobutrazol (F) | 22.471 | 236 > 125 (10) | 125 > 89 (20) | 236 > 167 (10) |
|  | Endosulfan alpha (I) | 22.522 | 195 > 159 (5) | 195 > 160 (5) | 195 > 125 (20) |
|  | Tetrachlorvinfos (I) | 22.885 | 329 > 109 (15) | 331 > 109 (15) | 109 > 79 (5) |
|  | Mepanipirym (F) | 23.004 | 223 > 222 (10) | 222 > 207 (15) | 221 > 220 (15) |
|  | Flutriafol (F) | 23.095 | 123 > 95 (15) | 123 > 75 (25) | 219 > 123 (15) |
|  | Napropamide (H) | 23.375 | 128 > 72 (5) | 128 > 100 (10) | 271 > 72 (15) |
|  | Hexaconazole (F) | 23.441 | 256 > 82 (10) | 231 > 175 (10) | 256 > 159 (15) |
|  | Picoxystrobin (F) | 23.720 | 145 > 102 (25) | 145 > 115 (15) | 145 > 117 (10) |
|  | Flutolanil (F) | 23.764 | 173 > 145 (15) | 281 > 173 (10) | 173 > 95 (30) |
|  | Imazalil (F) | 23.764 | 215 > 173 (5) | 217 > 175 (5) | 173 > 145 (15) |
|  | Dieldrin (I) | 23.778 | 277 > 241 (5) | 263 > 193 (35) | 263 > 191 (35) |
|  | Isoprothiolane (F) | 23.816 | 162 > 85 (20) | 162 > 134 (5) | 231 > 189 (10) |
|  | Profenofos (I) | 23.848 | 208 > 63 (30) | 339 >269 (15 ) | 299 > 269 (5) |
|  | p,p’ DDE (I) | 23.959 | 246 > 176 (30) | 316 > 246 (15) | 318 > 246 (15) |
|  | Fludioxonil (F) | 24.112 | 248 > 154 (20) | 248 > 182 (10) | 248 > 127 (30) |
|  | Myclobutanyl (F) | 24.390 | 179 > 125 (10) | 179 > 90(30) | 150 > 123 (15) |
|  | Prothioconazole (F) | 24.422 | 186 > 53 (20) | 186 > 117 (10) | 186 > 89 (10) |
|  | Iprowalikarb (F) | 24.468, 24.878 | 119 > 91 (15) | 116 > 98 (5) | 116 > 55 (15) |
|  | Azaconazole (F) | 24.482 | 217 > 173 (15) | 219 > 175 (15) | 173 > 145 (15) |
|  | Buprofezin (I) | 24.496 | 105 > 104 (10) | 105 > 77 (20) | 119 > 91 (15) |
|  | Metamitron (H) | 24.496 | 104 > 77 (15) | 202 > 174 (5) | 104 > 51 (35) |
|  | Flusilazole (F) | 24.550 | 233 > 165 (15) | 233 > 91 (20) | 315 > 233 (10) |
|  | Oxyflurofen (H) | 24.666 | 252 > 196 (20) | 252 > 146 (30) | 300 > 223 (15) |
|  | Endrin (I) | 24.678 | 263 > 193 (35) | 245 > 173 (30) | 317 > 281 (5) |
|  | Bupirimate (F) | 24.754 | 273 > 193 (5) | 273 > 108 (15) | 208 > 165 (10) |
|  | Nitrofen (H) | 24.808 | 202 > 139 (20) | 283 > 253 (10) | 283 > 202 (10) |
|  | Kresoxim-methyl (F) | 24.845 | 116 > 89 (15) | 116 > 63 (30) | 131 > 89 (30) |
|  | Cyproconazole (I) | 24.849 | 139 > 111 (15) | 222 > 125 (15) | 222 > 82 (10) |
|  | Endosulfan beta (I) | 25.095 | 207 > 172 (15) | 195 > 159 (10) | 195 > 125 (25) |
|  | Cyflufenamid (F) | 25.212 | 118 > 90 (10) | 118 > 89 (25) | 188 > 88 ( 35) |
|  | Fluazifop-p-butyl (H) | 25.288 | 282 > 91 (20) | 282 > 238 (20) | 383 > 282 (10) |
|  | Dinikonazol (F) | 25.524 | 268 > 232 (10) | 270 > 232 (10) | 270 > 234 (10) |
|  | Etaconazole (F) | 25.571 | 173 > 145 (15) | 173 > 109 (30) | 245 > 55 (10) |
|  | p,p’ DDD (I) | 25.650 | 235 > 165 (20) | 237 > 165 (20) | 235 > 199 (15) |
|  | o,p’ DDT (I) | 25.729 | 235 > 165 (20) | 237 > 165 (20) | 235 > 199 (15) |
|  | Oksadiksyl (F) | 25.842 | 163 > 132 (5) | 163 > 117 (25) | 132 > 117 (15) |
|  | Ethion (I) | 25.958 | 153 > 97 (10) | 125 > 97 (10) | 231 > 175 (10) |
|  | Triazophos (I) | 26.431 | 161 > 134 (5) | 161 > 106 (10) | 161 > 91 (15) |
|  | Benalaxyl (F) | 26.700 | 148 > 105 (20) | 148 > 77 (35 ) | 266 > 148 (5) |
|  | Endosulfan sulphate (I) | 26.732 | 272 > 237 (15) | 274 > 239 (15) | 274 > 237 (15) |
|  | Quinoxyfen (F) | 26.740 | 237 > 208 (30) | 272 > 237 (10) | 307 > 237 (20) |
|  | Lenacil (H) | 26.870 | 153 > 136 (15) | 153 > 82 (20) | 153 > 110 (15) |
|  | Fenhexamid (F) | 26.885 | 97 > 55 (10) | 177 > 78 (25) | 177 > 113 (15) |
|  | Propiconazole (F) | 26.889, 27.101 | 173 > 145 (15) | 173 > 109 (30) | 173 > 74 (45) |
|  | p.p’ DDT (I) | 26.959 | 235 > 165 (20) | 237 > 165 (20) | 235 > 199 (15) |
|  | Chloridazon (H) | 27.078 | 223 > 77 (15) | 221 > 220 (5 ) | 221 > 77 (20) |
|  | Trifloxystrobin (F) | 27.282 | 116 > 89 (15) | 172 > 145 (15) | 116 > 63 (30) |
|  | Fluopicolide (F) | 27.326 | 209 > 182 (10) | 209 > 146 (20) | 347 > 172 (20) |
|  | Tebuconazole (F) | 27.418 | 125 > 89 (15) | 250 > 125 (20) | 125 > 99 (20) |
|  | Captafol (F) | 27.584 | 150 > 79 (5) | 183 > 79 (10) | 150 > 72 (5) |
|  | Propargite (A) | 27.712 | 135 > 107 (10) | 150 > 135 (5) | 135 > 77 (30) |
|  | Diflufenican (DFF) (H) | 27.778 | 266 > 238 (15) | 266 > 246 (15) | 394 > 266 (10) |
|  | Epoxiconazole (F) | 28.005 | 192 > 138 (10) | 192 > 111 (25) | 165 > 138 (10) |
|  | Zoxamide (F) | 28.036 | 187 > 159 (15) | 189 > 161 (15) | 187 > 123 (30) |
|  | Iprodione (F) | 28.408 | 244 > 187 (5) | 187 > 124 (25) | 314 > 56 (20) |
|  | Bromuconazole (F) | 28.429, 29.171 | 173 > 145 (15) | 173 > 109 (30) | 175 > 147 (15) |
|  | Phosmet (I) | 28.489 | 160 > 77 (20) | 160 > 133 (10) | 160 > 105 (15) |
|  | Acetamiprid (I) | 28.509 | 152 > 116 (15) | 126 > 90 (5) | 126 > 99 (10) |
|  | Tetramethrin (I) | 28.591, 28.811 | 164 >107 (10) | 164 > 77 (25) | 123 > 81 (10) |
|  | Bromopropylate (A) | 28.619 | 183 > 155 (15) | 185 > 157 (15) | 339 > 183 (20) |
|  | EPN (I) | 28.626 | 169 > 141 (5) | 169 > 77 (25) | 185 > 157 (5) |
|  | Carbosulfan (I) | 28.660 | 164 > 149 (10) | 118 > 76 (5) | 164 > 103 (25) |
|  | Fenoxycarb (I) | 28.691 | 255 > 186 (10) | 186 > 158 (5) | 185 > 129. (5) |
|  | Dimoksystrobina (F) | 28.713 | 116 > 89 (15) | 116 > 63 (30) | 205 > 116 (10) |
|  | Chlorantraniliprole (I) | 28.809 | 239 > 214 (20) | 277 > 243 (10) | 277 >250 (10) |
|  | Bifenthrin (I) | 28.836 | 181 > 166 (10) | 181 > 165 (25) | 166 > 165 (20) |
|  | Methoxychlor (DMDT) (I) | 28.859 | 227 > 169 (25) | 227 > 141 (40) | 227 > 212 (15) |
|  | Bifenazat (I) | 28.879 | 184 > 156 (10) | 199 > 184 (10) | 184 > 169 (10) |
|  | Fenpropathrin (I) | 28.987 | 181 > 152 (25) | 208 > 181 (5) | 125 > 55 (10) |
|  | Etoxazol (A) | 29.057 | 141 > 113 (15) | 141 > 63 (30) | 204 > 176 (10) |
|  | Metconazol (F) | 29.067 | 125 > 89 (20) | 125 > 99 (20) | 138 > 69 (10) |
|  | Fenamidon (F) | 29.072 | 238 > 237 (10) | 268 > 180 (20) | 238 > 103 (15) |
|  | Tebufenpyrad (A) | 29.075 | 276 > 171 (10) | 333 > 171 (15) | 318 > 131 (15) |
|  | Fenazaquin (A) | 29.093 | 145 > 117 (10) | 160 > 145 (5) | 160 > 117 (20) |
|  | Tetradifon (I) | 29.381 | 159 > 131 (10) | 227 > 199 (15) | 159 > 111 (20) |
|  | Triticonazole (F) | 29.550 | 235 > 217 (5) | 235 > 182 (10) | 217 > 165 (25) |
|  | Azinphos methyl (I) | 29.648 | 160 > 132 (10) | 132 > 77 (15) | 160 > 77 (20) |
|  | Flurtamone (H) | 29.656 | 333 > 120 (15) | 199 > 157 (20) | 157 > 137 (15) |
|  | Phosalone (I) | 29.676 | 182 > 111 (15) | 182 > 102 (15) | 182 > 75 (30) |
|  | Pyriproxyfen (I) | 29.864 | 136 > 78 (20) | 136 > 96 (15) | 321 > 222 (10) |
|  | Lambda cyhalothrin (I) | 30.099, 30.398 | 197 > 141 (10) | 181 > 152 (25) | 197 > 161 (5) |
|  | Amitraz (I) | 30.161 | 132 > 117 (15) | 162 > 132 (5) | 162 > 121 (10) |
|  | Acrinathrin (I) | 30.389, 30.713 | 207 > 181 (10) | 181 > 152 (30) | 289 > 93 (10) |
|  | Fenarimol (F) | 30.416 | 219 > 107 (10) | 251 > 139 (10) | 139 > 75 (30) |
|  | Azinphos ethyl (I) | 30.653 | 132 > 77 (15) | 160 > 77 (20) | 160 > 132 (10) |
|  | Pyrazofos (I) | 30.685 | 221 > 193 (10) | 232 > 204 (10) | 221 > 149 (15) |
|  | Metrafenone (F) | 30.918 | 209 > 166 (10) | 395 > 365 (15) | 227 > 169 (10) |
|  | Fenoxaprop-P-ethyl (H) | 30.970 | 361 > 288 (10) | 288 > 91 (20) | 288 > 119 (10) |
|  | Bitertanol (F) | 31.254 | 170 > 141 (20) | 170 > 115 (40) | 168 > 70 (10) |
|  | Spirodiclofen (A) | 31.348 | 109 > 81 (10) | 109 > 79 (15) | 312 > 259 (10) |
|  | Permethrin (I) | 31.385, 31.574 | 183 > 168 (10) | 183 > 165 (10) | 183 > 153 (15) |
|  | Pyridaben (A) | 31.539 | 147 > 117 (20) | 147 > 132 (10) | 147 > 105 (10) |
|  | Fluquinconazole (F) | 31.654 | 340 > 298 (15) | 108 > 57 (15) | 340 > 108 (40) |
|  | Prochloraz (F) | 31.765 | 196 > 97 (30) | 180 > 138 (10) | 310 > 70 (15) |
|  | Fenbuconazole (F) | 32.205 | 129 > 102 (15) | 198 > 129 (5) | 129 > 78 (20) |
|  | Cyfluthrin (I) | 32.266, 32.413  32.678, 32.741 | 163 > 91 (15) | 163 > 127 (5 ) | 199 > 170 (25) |
|  | Cypermethrin (I) | 32.739, 33.935  33.023, 33.086 | 163 > 91 (10) | 163 > 127 (5) | 165 > 91 (10) |
|  | Boscalid (F) | 32.775 | 140 > 112 (10) | 140 > 76 (25) | 112 > 76 (15) |
|  | Quizalofop-P-ethyl (H) | 32.957 | 372 > 299 (10) | 163 > 136 (10) | 163 > 100 (20) |
|  | Esfenvalerate/Fenvalerate (I) | 34.342, 34.762 | 167 > 125 (5) | 209 > 141 (15) | 181 > 152 (20) |
|  | Pyraklostrobina (F) | 34.449 | 132 >77.1 (20) | 164 > 132 (10) | 132 > 104 (15) |
|  | Tau fluvalinate (I) | 34.765, 34.900 | 250 > 55 (40) | 181 > 152 (40) | 250 > 200 (40) |
|  | Difenoconazole (F) | 35.166, 35.317 | 323 > 265 (15) | 265 > 202 (20) | 325 > 267 (15) |
|  | Deltamethrin (I) | 35.463, 35.967 | 253 > 93 (15) | 181 > 152 (25) | 251 > 172 (5) |
|  | Indoxacarb (I) | 35.885 | 203 > 134 (15) | 203 > 106 (25) | 203 > 78 (30) |
|  | Azoxystrobin (F) | 36.647 | 344 > 329 (15) | 344 > 172 (40) | 344 > 183 (25) |
|  | Famoksadone (F) | 36.697 | 197 > 141 (15) | 224 > 196 (10) | 197 > 115 (30) |
|  | Dimethomorph (F) | 36.705, 37.705 | 301 > 165 (10) | 303 > 165 (10) | 387 > 301 (10) |
|  | Imibenkonazol (F) | 38.115 | 125 > 89 (20) | 125 > 99 (20) | 253 > 82 (5) |
|  | Propaquizafop (H) | 40.523 | 163 > 100 (25) | 163 > 136 (10) | 299 > 91 (20) |
|  | Triphenyl phosphate (TPP) (IS) | 27.740 | 215 > 168 (15) | 232 > 215 (10) | 326 > 325 (5) |

*t_R_*– retention time, H – Herbicide, F– Fungicide, I – Insecticide, A – Acaricide.
