# Supplementary material for: Influence of QuEChERS modifications on recovery and matrix effect during the multi-residue pesticide analysis in soil by GC/MS/MS and GC/ECD/NPD
Source: Environ Sci Pollut Res Int. 2017 Jan 16;24(8):7124–38. doi: 10.1007/s11356-016-8334-1 (PMC5383684; doi:10.1007/s11356-016-8334-1)
Supplement: Supplementary file 2 — Matrix effects, linear range, correlation coefficients, recoveries, RSDs and expanded uncertainties (U) of 216 pesticides QuEChERS method without cleanup step analysis by GC-μECD-NPD and GC/MS/MS. (DOCX 73 kb.) [file 11356_2016_8334_MOESM2_ESM.docx]

**Table S2.** Matrix effects, linear range, correlation coefficients, recoveries, RSDs and expanded uncertainties (U) of 216 pesticides QuEChERS method without clean-up step analysis by GC-µECD-NPD and GC-MS/MS.

| No. of active substance | ID of pesticides | Range (mg kg^-1^) | R^2^ | ME (%) | U (%) | Recovery (%)  LOQ | RSD (%) | | Recovery (%)  10 LOQ | | | RSD (%) | Recovery (%)  100 LOQ | | RSD (%) | | Range (mg kg^-1^) | R^2^ | ME (%) | U (%) | Recovery (%)  LOQ | RSD (%) | Recovery (%)  10 LOQ | RSD (%) | Recovery (%)  100 LOQ | RSD (%) |
| --- | --- | --- | --- | --- | --- | --- | --- | --- | --- | --- | --- | --- | --- | --- | --- | --- | --- | --- | --- | --- | --- | --- | --- | --- | --- | --- |
|  |  | GC µECD-NPD | | | | | | | | | | | | | | | GC-MS/MS | | | | | | | | | |
|  | 134 | 0.03-3.0 | 0.999 | 18 | 21 | 62 | | 18 | | 65 | 15 | | | 60 | | 12 | 0.005-0.5 | 0.998 | 3 | 21 | 79 | 1 | 80 | 1 | 78 | 6 |
|  | 50 | 0.01-1.0 | 0.999 | 9 | 17 | 65 | | 1 | | 67 | 1 | | | 66 | | 1 | 0.005-0.5 | 0.999 | 15 | 24 | 78 | 4 | 75 | 4 | 70 | 2 |
|  | 48 | 0.01-1.0 | 0.999 | -3 | 18 | 62 | | 3 | | 65 | 1 | | | 60 | | 1 | 0.005-0.5 | 0.999 | -14 | 23 | 84 | 3 | 83 | 3 | 89 | 3 |
|  | 141 | 0.01-1.0 | 0.999 | -2 | 21 | 92 | | 3 | | 87 | 2 | | | 95 | | 4 | 0.005-0.5 | 0.999 | 12 | 24 | 77 | 4 | 70 | 4 | 70 | 9 |
|  | 177 | 0.02-2.0 | 0.999 | -8 | 21 | 61 | | 3 | | 68 | 1 | | | 71 | | 3 | 0.005-0.5 | 0.999 | -12 | 15 | 92 | 7 | 79 | 7 | 82 | 3 |
|  | 133 | 0.01-1.0 | 0.999 | -20 | 17 | 60 | | 1 | | 61 | 1 | | | 62 | | 1 | 0.01-1.0 | 0.999 | -4 | 23 | 82 | 3 | 84 | 3 | 78 | 2 |
|  | 147 | 0.05-5.0 | 0.999 | 13 | 25 | 62 | | 10 | | 60 | 14 | | | 59 | | 15 | 0.005-0.5 | 0.994 | 5 | 27 | 81 | 7 | 95 | 7 | 90 | 5 |
|  | 120 | 0.01-1.0 | 0.999 | 47 | 25 | 105 | | 7 | | 110 | 7 | | | 100 | | 9 | 0.005-0.5 | 0.999 | 48 | 28 | 107 | 8 | 120 | 8 | 120 | 1 |
|  | 108 | 0.01-1.0 | 0.999 | -4 | 20 | 80 | | 17 | | 78 | 3 | | | 90 | | 3 | 0.005-0.5 | 0.999 | -8 | 18 | 102 | 9 | 115 | 9 | 118 | 5 |
|  | 44 | 0.02-2.0 | 0.998 | -3 | 16 | 82 | | 5 | | 73 | 3 | | | 82 | | 6 | 0.005-0.5 | 0.999 | -6 | 21 | 70 | 1 | 70 | 1 | 69 | 6 |
|  | 196 | 0.01-1.0 | 0.999 | 18 | 18 | 67 | | 2 | | 63 | 1 | | | 70 | | 2 | 0.005-0.5 | 0.997 | 1 | 27 | 80 | 12 | 103 | 12 | 97 | 3 |
|  | 88 | 0.01-1.0 | 0.999 | 12 | 22 | 90 | | 12 | | 85 | 8 | | | 65 | | 13 | 0.005-0.5 | 0.997 | 11 | 27 | 70 | 12 | 75 | 12 | 93 | 7 |
|  | 179 | 0.01-1.0 | 0.999 | 31 | 30 | 115 | | 14 | | 95 | 13 | | | 110 | | 14 | 0.005-0.5 | 0.997 | 24 | 24 | 93 | 9 | 75 | 9 | 88 | 4 |
|  | 173 | 0.01-1.0 | 0.999 | 24 | 21 | 75 | | 4 | | 80 | 4 | | | 85 | | 2 | 0.005-0.5 | 0.999 | 28 | 28 | 73 | 13 | 91 | 5 | 99 | 6 |
|  | 62 | 0.01-1.0 | 0.999 | 0 | 16 | 58 | | 2 | | 62 | 3 | | | 60 | | 1 | 0.005-0.5 | 0.999 | 37 | 25 | 72 | 15 | 96 | 6 | 100 | 1 |
|  | 72 | 0.01-1.0 | 0.999 | -18 | 18 | 95 | | 3 | | 88 | 4 | | | 96 | | 4 | 0.005-0.5 | 0.999 | 6 | 21 | 70 | 6 | 72 | 15 | 81 | 3 |
|  | 33 | 0.01-1.0 | 0.999 | -2 | 19 | 72 | | 3 | | 84 | 3 | | | 86 | | 2 | 0.005-0.5 | 0.999 | -7 | 19 | 98 | 4 | 103 | 4 | 105 | 0 |
|  | 53 | 0.01-1.0 | 0.998 | 38 | 24 | 106 | | 6 | | 96 | 9 | | | 105 | | 8 | 0.005-0.5 | 0.997 | 5 | 24 | 80 | 9 | 96 | 4 | 80 | 9 |
|  | 158 | 0.02-2.0 | 0.999 | 53 | 17 | 104 | | 16 | | 100 | 4 | | | 106 | | 4 | 0.01-1.0 | 0.995 | 10 | 20 | 81 | 5 | 86 | 4 | 90 | 2 |
|  | 213 | 0.01-1.0 | 0.999 | 20 | 17 | 75 | | 1 | | 76 | 1 | | | 73 | | 1 | 0.005-0.5 | 0.995 | 3 | 21 | 72 | 6 | 83 | 6 | 73 | 2 |
|  | 23 | 0.01-1.0 | 0.999 | -7 | 23 | 98 | | 3 | | 93 | 6 | | | 110 | | 5 | 0.005-0.5 | 0.999 | -16 | 10 | 88 | 1 | 87 | 2 | 89 | 8 |
|  | 162 | 0.01-1.0 | 0.999 | -17 | 16 | 64 | | 11 | | 67 | 1 | | | 69 | | 1 | 0.005-0.5 | 0.999 | -7 | 21 | 76 | 6 | 65 | 3 | 68 | 1 |
|  | 103 | 0.005-0.5 | 0.995 | -4 | 19 | 82 | | 6 | | 89 | 6 | | | 95 | | 4 | 0.005-0.5 | 0.999 | -4 | 19 | 71 | 4 | 71 | 5 | 78 | 4 |
|  | 109 | 0.005-0.5 | 0.999 | 3 | 20 | 70 | | 9 | | 71 | 10 | | | 79 | | 10 | 0.005-0.5 | 0.999 | 3 | 18 | 72 | 3 | 77 | 4 | 76 | 5 |
|  | 46 | 0.03-3.0 | 0.998 | -19 | 19 | 86 | | 4 | | 94 | 4 | | | 86 | | 4 | 0.01-1.0 | 0.999 | -11 | 17 | 74 | 2 | 70 | 4 | 70 | 4 |
|  | 51 | 0.005-0.5 | 0.988 | 82 | 17 | 93 | | 2 | | 87 | 3 | | | 90 | | 3 | 0.005-0.5 | 0.994 | 3 | 25 | 88 | 10 | 85 | 6 | 104 | 2 |
|  | 58 | 0.01-0.5 | 0.999 | 9 | 20 | 97 | | 5 | | 92 | 2 | | | 98 | | 6 | 0.005-0.5 | 0.997 | 7 | 19 | 109 | 4 | 116 | 4 | 116 | 5 |
|  | 191 | 0.01-1.0 | 0.999 | -12 | 19 | 95 | | 2 | | 89 | 3 | | | 98 | | 1 | 0.005-0.5 | 0.999 | 1 | 16 | 100 | 1 | 101 | 1 | 101 | 4 |
|  | 27 | 0.02-2.0 | 0.999 | 84 | 29 | 73 | | 9 | | 70 | 15 | | | 76 | | 4 | 0.005-0.5 | 0.999 | 1 | 19 | 84 | 6 | 79 | 6 | 73 | 5 |
|  | 6 | 0.01-0.5 | 0.999 | -4 | 19 | 92 | | 4 | | 90 | 1 | | | 98 | | 2 | 0.005-1.0 | 0.999 | -1 | 18 | 79 | 3 | 81 | 1 | 76 | 4 |
|  | 36 | 0.03-3.0 | 0.999 | -5 | 19 | 102 | | 4 | | 93 | 3 | | | 92 | | 5 | 0.005-0.5 | 0.999 | 21 | 22 | 101 | 7 | 88 | 2 | 90 | 4 |
|  | 104 | 0.005-0.5 | 0.999 | -11 | 19 | 90 | | 11 | | 94 | 12 | | | 106 | | 10 | 0.005-0.5 | 0.999 | -7 | 24 | 95 | 9 | 78 | 1 | 82 | 4 |
|  | 176 | 0.01-1.0 | 0.999 | 45 | 17 | 102 | | 1 | | 95 | 11 | | | 103 | | 3 | 0.005-0.5 | 0.999 | 6 | 21 | 94 | 6 | 83 | 2 | 85 | 2 |
|  | 105 | 0.005-0.5 | 0.991 | -9 | 16 | 92 | | 10 | | 90 | 11 | | | 102 | | 9 | 0.005-0.5 | 0.999 | -12 | 18 | 90 | 5 | 98 | 3 | 89 | 1 |
|  | 198 | 0.01-1.0 | 0.999 | -15 | 17 | 90 | | 1 | | 90 | 1 | | | 103 | | 9 | 0.005-0.5 | 0.999 | 1 | 23 | 90 | 6 | 90 | 8 | 80 | 2 |
|  | 180 | 0.02-2.0 | 0.999 | 23 | 20 | 106 | | 3 | | 96 | 4 | | | 105 | | 5 | 0.005-0.5 | 0.999 | 16 | 18 | 119 | 6 | 108 | 3 | 115 | 5 |
|  | 185 | 0.01-1.0 | 0.997 | -6 | 19 | 90 | | 7 | | 84 | 7 | | | 95 | | 5 | 0.005-0.5 | 0.999 | 9 | 17 | 98 | 7 | 88 | 2 | 85 | 4 |
|  | 47 | 0.01-1.0 | 0.999 | -7 | 17 | 89 | | 2 | | 85 | 1 | | | 89 | | 2 | 0.005-0.5 | 0.999 | 4 | 18 | 97 | 6 | 85 | 3 | 90 | 2 |
|  | 154 | 0.01-1.0 | 0.999 | 41 | 27 | 103 | | 12 | | 94 | 10 | | | 103 | | 9 | 0.005-0.5 | 0.995 | 0 | 27 | 118 | 5 | 119 | 2 | 110 | 2 |
|  | 32 | 0.01-1.0 | 0.999 | 55 | 18 | 74 | | 9 | | 64 | 4 | | | 68 | | 6 | 0.005-0.5 | 0.999 | 1 | 20 | 68 | 6 | 78 | 5 | 69 | 3 |
|  | 197 | 0.01-1.0 | 0.999 | 9 | 20 | 98 | | 12 | | 86 | 12 | | | 98 | | 3 | 0.005-0.5 | 0.999 | 14 | 16 | 94 | 7 | 80 | 1 | 85 | 1 |
|  | 100 | 0.01-1.0 | 0.999 | 21 | 17 | 86 | | 8 | | 91 | 8 | | | 82 | | 7 | 0.005-0.5 | 0.997 | 2 | 20 | 80 | 6 | 80 | 5 | 90 | 6 |
|  | 137 | 0.05-5.0 | 0.999 | 14 | 23 | 79 | | 14 | | 73 | 6 | | | 72 | | 1 | 0.005-0.5 | 0.999 | 23 | 15 | 75 | 6 | 71 | 6 | 82 | 4 |
|  | 166 | 0.01-1.0 | 0.999 | -10 | 22 | 88 | | 3 | | 85 | 2 | | | 96 | | 2 | 0.005-0.5 | 0.999 | 7 | 25 | 102 | 7 | 96 | 13 | 88 | 3 |
|  | 41 | 0.01-1.0 | 0.999 | 7 | 21 | 102 | | 12 | | 98 | 1 | | | 99 | | 2 | 0.005-0.5 | 0.999 | 12 | 14 | 88 | 2 | 89 | 2 | 86 | 2 |
|  | 57 | 0.02-2.0 | 0.999 | 15 | 17 | 90 | | 6 | | 84 | 4 | | | 106 | | 9 | 0.01-1.0 | 0.999 | 15 | 15 | 85 | 4 | 78 | 3 | 80 | 6 |
|  | 140 | 0.01-1.0 | 0.999 | 14 | 22 | 105 | | 12 | | 85 | 3 | | | 95 | | 4 | 0.005-0.5 | 0.999 | 4 | 13 | 95 | 7 | 93 | 1 | 82 | 3 |
|  | 2 | 0.02-2.0 | 0.999 | 11 | 21 | 96 | | 2 | | 95 | 1 | | | 104 | | 1 | 0.005-0.5 | 0.999 | 3 | 15 | 86 | 4 | 91 | 3 | 84 | 2 |
|  | 102 | 0.02-2.0 | 0.999 | 84 | 23 | 75 | | 16 | | 66 | 5 | | | 68 | | 9 | 0.005-0.5 | 0.998 | 12 | 28 | 95 | 14 | 109 | 8 | 82 | 4 |
|  | 156 | 0.01-1.0 | 0.999 | 18 | 22 | 90 | | 13 | | 88 | 2 | | | 93 | | 1 | 0.005-0.5 | 0.998 | 14 | 22 | 76 | 13 | 84 | 10 | 102 | 3 |
|  | 35 | 0.005-0.5 | 0.999 | 7 | 22 | 89 | | 3 | | 85 | 2 | | | 89 | | 2 | 0.005-0.5 | 0.999 | 2 | 13 | 90 | 2 | 88 | 1 | 86 | 3 |
|  | 215 | 0.01-1.0 | 0.999 | 21 | 20 | 86 | | 11 | | 84 | 1 | | | 83 | | 2 | 0.005-0.5 | 0.999 | 2 | 15 | 84 | 6 | 95 | 3 | 85 | 1 |
|  | 106 | 0.005-0.5 | 0.999 | -6 | 20 | 98 | | 1 | | 91 | 10 | | | 80 | | 8 | 0.005-0.5 | 0.999 | -4 | 22 | 94 | 8 | 89 | 10 | 79 | 1 |
|  | 206 | 0.01-1.0 | 0.999 | 8 | 21 | 87 | | 2 | | 86 | 1 | | | 90 | | 1 | 0.005-0.5 | 0.999 | 12 | 14 | 73 | 10 | 90 | 2 | 90 | 2 |
|  | 26 | 0.05-2.5 | 0.998 | -1 | 19 | 63 | | 10 | | 67 | 9 | | | 70 | | 6 | 0.005-0.5 | 0.995 | -6 | 15 | 68 | 6 | 79 | 4 | 77 | 8 |
|  | 125 | 0.01-1.0 | 0.999 | -7 | 23 | 90 | | 15 | | 88 | 13 | | | 116 | | 16 | 0.005-0.5 | 0.995 | 1 | 27 | 119 | 9 | 102 | 5 | 114 | 2 |
|  | 79 | 0.01-1.0 | 0.999 | -4 | 21 | 66 | | 2 | | 61 | 2 | | | 67 | | 2 | 0.005-0.5 | 0.999 | 5 | 14 | 78 | 4 | 70 | 2 | 74 | 2 |
|  | 129 | 0.01-1.0 | 0.999 | -8 | 20 | 94 | | 11 | | 88 | 3 | | | 94 | | 2 | 0.01-1.0 | 0.999 | -2 | 15 | 80 | 5 | 90 | 3 | 88 | 1 |
|  | 153 | 0.01-1.0 | 0.999 | 14 | 21 | 89 | | 4 | | 89 | 1 | | | 89 | | 0 | 0.005-0.5 | 0.995 | 3 | 24 | 80 | 5 | 88 | 12 | 90 | 2 |
|  | 172 | 0.01-1.0 | 0.999 | -7 | 23 | 90 | | 3 | | 88 | 5 | | | 94 | | 1 | 0.005-0.5 | 0.999 | 3 | 15 | 90 | 6 | 84 | 3 | 78 | 4 |
|  | 199 | 0.01-1.0 | 0.999 | -12 | 20 | 86 | | 13 | | 85 | 1 | | | 91 | | 1 | 0.005-0.5 | 0.999 | -18 | 13 | 101 | 9 | 115 | 1 | 118 | 1 |
|  | 81 | 0.01-1.0 | 0.999 | -6 | 22 | 91 | | 3 | | 87 | 1 | | | 94 | | 3 | 0.005-0.5 | 0.999 | 2 | 16 | 80 | 6 | 90 | 4 | 80 | 3 |
|  | 188 | 0.01-1.0 | 0.999 | 7 | 17 | 85 | | 9 | | 80 | 10 | | | 85 | | 2 | 0.01-1.0 | 0.998 | 30 | 14 | 90 | 7 | 101 | 2 | 88 | 6 |
|  | 168 | 0.01-1.0 | 0.999 | -8 | 21 | 89 | | 1 | | 88 | 1 | | | 91 | | 1 | 0.005-0.5 | 0.999 | 1 | 13 | 93 | 4 | 85 | 1 | 90 | 2 |
|  | 16 | 0.01-1.0 | 0.999 | 55 | 20 | 83 | | 4 | | 94 | 6 | | | 91 | | 6 | 0.005-1.0 | 0.998 | 59 | 14 | 80 | 8 | 86 | 2 | 71 | 1 |
|  | 49 | 0.01-1.0 | 0.999 | -30 | 21 | 69 | | 3 | | 65 | 2 | | | 67 | | 2 | 0.005-0.5 | 0.998 | -21 | 14 | 60 | 5 | 68 | 2 | 68 | 2 |
|  | 4 | 0.005-0.5 | 0.999 | 2 | 18 | 83 | | 14 | | 90 | 6 | | | 92 | | 14 | 0.005-1.0 | 0.999 | 1 | 14 | 80 | 6 | 83 | 2 | 92 | 7 |
|  | 126 | 0.01-1.0 | 0.999 | -1 | 23 | 93 | | 5 | | 86 | 3 | | | 96 | | 3 | 0.005-0.5 | 0.998 | 11 | 15 | 108 | 2 | 110 | 3 | 106 | 4 |
|  | 138 | 0.01-1.0 | 0.999 | 4 | 22 | 97 | | 4 | | 88 | 3 | | | 106 | | 5 | 0.005-0.5 | 0.999 | 15 | 13 | 81 | 6 | 90 | 1 | 80 | 3 |
|  | 86 | 0.01-1.0 | 0.999 | -7 | 23 | 90 | | 13 | | 78 | 2 | | | 90 | | 3 | 0.005-0.5 | 0.999 | -7 | 13 | 80 | 10 | 83 | 1 | 99 | 4 |
|  | 52 | 0.005-0.5 | 0.999 | 45 | 22 | 98 | | 6 | | 91 | 4 | | | 108 | | 2 | 0.005-0.5 | 0.999 | 56 | 15 | 80 | 5 | 85 | 3 | 90 | 3 |
|  | 34 | 0.005-0.5 | 0.999 | 5 | 22 | 89 | | 2 | | 87 | 2 | | | 90 | | 1 | 0.005-0.5 | 0.999 | 8 | 15 | 80 | 3 | 86 | 3 | 86 | 3 |
|  | 85 | 0.02-2.0 | 0.998 | -10 | 23 | 98 | | 6 | | 94 | 2 | | | 102 | | 3 | 0.01-1.0 | 0.999 | -16 | 15 | 103 | 6 | 110 | 3 | 97 | 4 |
|  | 155 | 0.01-1.0 | 0.999 | 14 | 20 | 98 | | 1 | | 89 | 1 | | | 90 | | 1 | 0.005-0.5 | 0.999 | 3 | 17 | 70 | 8 | 76 | 5 | 86 | 1 |
|  | 37 | 0.01-1.0 | 0.999 | 21 | 22 | 92 | | 3 | | 101 | 4 | | | 108 | | 4 | 0.005-0.5 | 0.998 | 1 | 14 | 79 | 10 | 86 | 2 | 99 | 3 |
|  | 280 | 0.01-1.0 | 0.999 | -6 | 21 | 89 | | 11 | | 87 | 4 | | | 87 | | 1 | 0.005-0.5 | 0.999 | -4 | 14 | 90 | 7 | 85 | 2 | 98 | 2 |
|  | 117 | 0.01-1.0 | 0.999 | -27 | 16 | 101 | | 6 | | 95 | 6 | | | 108 | | 5 | 0.005-0.5 | 0.997 | 17 | 21 | 86 | 10 | 84 | 12 | 102 | 5 |
|  | 91 | 0.01-1.0 | 0.999 | 94 | 17 | 98 | | 3 | | 93 | 4 | | | 91 | | 1 | 0.005-0.5 | 0.999 | 23 | 13 | 80 | 7 | 90 | 1 | 76 | 6 |
|  | 201 | 0.01-1.0 | 0.999 | 14 | 16 | 96 | | 11 | | 89 | 1 | | | 91 | | 1 | 0.005-0.5 | 0.999 | 1 | 13 | 80 | 4 | 75 | 1 | 82 | 5 |
|  | 94 | 0.01-1.0 | 0.999 | 6 | 21 | 104 | | 4 | | 98 | 2 | | | 99 | | 3 | 0.005-0.5 | 0.998 | 3 | 10 | 80 | 12 | 82 | 1 | 102 | 2 |
|  | 18 | 0.01-1.0 | 0.999 | 10 | 20 | 92 | | 4 | | 84 | 1 | | | 88 | | 2 | 0.005-0.5 | 0.999 | 2 | 14 | 94 | 5 | 86 | 2 | 86 | 1 |
|  | 205 | 0.02-2.0 | 0.999 | -9 | 27 | 116 | | 11 | | 114 | 16 | | | 106 | | 15 | 0.005-0.5 | 0.999 | -8 | 13 | 72 | 6 | 73 | 1 | 82 | 6 |
|  | 101 | 0.01-1.0 | 0.999 | 83 | 19 | 110 | | 10 | | 108 | 9 | | | 90 | | 6 | 0.01-1.0 | 0.998 | 1 | 20 | 92 | 8 | 86 | 8 | 102 | 8 |
|  | 43 | 0.01-1.0 | 0.995 | -19 | 17 | 94 | | 5 | | 84 | 6 | | | 90 | | 7 | 0.005-0.5 | 0.999 | 14 | 13 | 90 | 6 | 89 | 1 | 80 | 6 |
|  | 167 | 0.01-1.0 | 0.999 | -9 | 22 | 91 | | 9 | | 89 | 2 | | | 90 | | 1 | 0.005-0.5 | 0.999 | 1 | 12 | 90 | 4 | 86 | 0 | 82 | 3 |
|  | 118 | 0.01-1.0 | 0.999 | -15 | 21 | 98 | | 1 | | 102 | 2 | | | 88 | | 2 | 0.005-0.5 | 0.999 | 16 | 12 | 101 | 13 | 80 | 0 | 76 | 2 |
|  | 131 | 0.01-1.0 | 0.999 | 11 | 23 | 110 | | 4 | | 104 | 4 | | | 98 | | 5 | 0.005-0.5 | 0.999 | 17 | 13 | 80 | 4 | 86 | 1 | 88 | 4 |
|  | 107 | 0.005-0.5 | 0.999 | 1 | 16 | 90 | | 15 | | 106 | 15 | | | 85 | | 9 | 0.005-0.5 | 0.999 | 0 | 15 | 102 | 12 | 86 | 3 | 80 | 5 |
|  | 159 | 0.01-1.0 | 0.999 | 15 | 22 | 97 | | 4 | | 95 | 6 | | | 98 | | 3 | 0.005-0.5 | 0.999 | 1 | 19 | 86 | 10 | 104 | 7 | 104 | 3 |
|  | 204 | 0.03-3.0 | 0.998 | 96 | 20 | 61 | | 4 | | 72 | 4 | | | 67 | | 3 | 0.005-0.5 | 0.995 | 65 | 15 | 69 | 1 | 69 | 3 | 68 | 1 |
|  | 157 | 0.01-1.0 | 0.999 | -14 | 16 | 84 | | 12 | | 90 | 2 | | | 92 | | 2 | 0.005-0.5 | 0.999 | -12 | 15 | 92 | 5 | 82 | 3 | 90 | 5 |
|  | 25 | 0.01-0.5 | 0.997 | 4 | 22 | 60 | | 1 | | 68 | 4 | | | 62 | | 5 | 0.005-0.5 | 0.995 | 0 | 17 | 79 | 5 | 78 | 5 | 70 | 1 |
|  | 207 | 0.01-1.0 | 0.999 | -4 | 21 | 60 | | 10 | | 69 | 8 | | | 74 | | 7 | 0.005-0.5 | 0.998 | -5 | 13 | 65 | 3 | 71 | 1 | 69 | 10 |
|  | 30 | 0.01-1.0 | 0.999 | 0 | 22 | 103 | | 4 | | 94 | 2 | | | 102 | | 3 | 0.005-0.5 | 0.999 | 7 | 15 | 109 | 6 | 116 | 3 | 105 | 3 |
|  | 99 | 0.01-1.0 | 0.997 | 7 | 24 | 63 | | 10 | | 68 | 1 | | | 70 | | 3 | 0.005-0.5 | 0.995 | 0 | 14 | 68 | 1 | 69 | 2 | 68 | 13 |
|  | 119 | 0.01-1.0 | 0.999 | -6 | 16 | 100 | | 6 | | 93 | 6 | | | 95 | | 4 | 0.005-0.5 | 0.999 | 11 | 20 | 83 | 7 | 91 | 8 | 78 | 5 |
|  | 187 | 0.01-1.0 | 0.999 | -14 | 22 | 99 | | 6 | | 96 | 3 | | | 90 | | 2 | 0.005-0.5 | 0.999 | 10 | 14 | 98 | 9 | 80 | 2 | 85 | 3 |
|  | 209 | 0.02-2.0 | 0.999 | -10 | 20 | 82 | | 2 | | 89 | 4 | | | 96 | | 5 | 0.01-1.0 | 0.999 | 14 | 14 | 86 | 7 | 73 | 2 | 75 | 1 |
|  | 161 | 0.01-1.0 | 0.999 | 13 | 20 | 89 | | 10 | | 86 | 2 | | | 92 | | 2 | 0.005-0.5 | 0.999 | 3 | 14 | 83 | 7 | 84 | 2 | 96 | 1 |
|  | 127 | 0.01-1.0 | 0.999 | -4 | 21 | 91 | | 4 | | 93 | 2 | | | 98 | | 2 | 0.005-0.5 | 0.999 | 19 | 15 | 88 | 7 | 82 | 3 | 96 | 2 |
|  | 87 | 0.005-0.5 | 0.998 | -32 | 20 | 90 | | 2 | | 95 | 2 | | | 98 | | 1 | 0.005-0.5 | 0.995 | -25 | 19 | 96 | 4 | 89 | 7 | 88 | 1 |
|  | 170 | 0.01-1.0 | 0.999 | 18 | 22 | 84 | | 12 | | 90 | 3 | | | 85 | | 3 | 0.005-0.5 | 0.999 | 31 | 14 | 86 | 11 | 72 | 2 | 94 | 3 |
|  | 111 | 0.05-5.0 | 0.999 | -2 | 21 | 90 | | 4 | | 98 | 3 | | | 88 | | 3 | 0.01-1.0 | 0.997 | -3 | 17 | 77 | 5 | 86 | 5 | 82 | 10 |
|  | 135 | 0.01-1.0 | 0.999 | -1 | 22 | 96 | | 6 | | 91 | 3 | | | 97 | | 3 | 0.005-0.5 | 0.999 | 18 | 13 | 80 | 1 | 81 | 1 | 81 | 3 |
|  | 212 | 0.01-1.0 | 0.999 | 30 | 22 | 88 | | 15 | | 88 | 4 | | | 98 | | 3 | 0.005-0.5 | 0.999 | 3 | 16 | 113 | 2 | 117 | 4 | 116 | 3 |
|  | 17 | 0.01-1.0 | 0.998 | 14 | 16 | 107 | | 3 | | 91 | 6 | | | 105 | | 4 | 0.005-1.0 | 0.999 | 1 | 13 | 87 | 9 | 79 | 1 | 70 | 5 |
|  | 152 | 0.02-2.0 | 0.999 | -12 | 20 | 93 | | 11 | | 84 | 2 | | | 91 | | 2 | 0.005-0.5 | 0.999 | -7 | 17 | 86 | 8 | 96 | 5 | 80 | 1 |
|  | 63 | 0.005-0.5 | 0.999 | 1 | 23 | 97 | | 3 | | 93 | 3 | | | 110 | | 2 | 0.005-0.5 | 0.999 | 0 | 15 | 103 | 11 | 86 | 3 | 82 | 4 |
|  | 200 | 0.01-1.0 | 0.999 | 7 | 21 | 108 | | 10 | | 90 | 6 | | | 100 | | 6 | 0.005-0.5 | 0.998 | 2 | 14 | 86 | 5 | 84 | 2 | 94 | 10 |
|  | 128 | 0.01-1.0 | 0.999 | -15 | 16 | 105 | | 5 | | 94 | 6 | | | 106 | | 3 | 0.005-0.5 | 0.998 | -19 | 12 | 71 | 6 | 77 | 0 | 82 | 5 |
|  | 98 | 0.01-1.0 | 0.999 | -8 | 20 | 92 | | 15 | | 90 | 1 | | | 93 | | 1 | 0.01-1.0 | 0.999 | -18 | 16 | 86 | 5 | 86 | 4 | 95 | 1 |
|  | 143 | 0.02-2.0 | 0.998 | -5 | 20 | 96 | | 2 | | 87 | 5 | | | 93 | | 6 | 0.005-0.5 | 0.999 | 15 | 14 | 102 | 2 | 105 | 2 | 106 | 1 |
|  | 110 | 0.01-1.0 | 0.999 | -19 | 16 | 100 | | 4 | | 93 | 4 | | | 106 | | 7 | 0.005-0.5 | 0.997 | -11 | 13 | 84 | 4 | 91 | 1 | 92 | 5 |
|  | 165 | 0.01-1.0 | 0.999 | 16 | 21 | 98 | | 7 | | 103 | 3 | | | 92 | | 2 | 0.01-1.0 | 0.999 | 38 | 16 | 104 | 12 | 90 | 4 | 80 | 2 |
|  | 97 | 0.02-2.0 | 0.999 | -13 | 20 | 93 | | 12 | | 94 | 1 | | | 93 | | 1 | 0.005-0.5 | 0.999 | -10 | 16 | 96 | 3 | 90 | 4 | 90 | 1 |
|  | 112 | 0.01-1.0 | 0.998 | 82 | 20 | 80 | | 8 | | 79 | 4 | | | 73 | | 2 | 0.005-0.5 | 0.999 | 74 | 14 | 88 | 2 | 84 | 2 | 86 | 1 |
|  | 54 | 0.005-0.5 | 0.999 | 2 | 21 | 103 | | 9 | | 96 | 14 | | | 85 | | 8 | 0.005-0.5 | 0.999 | 0 | 19 | 84 | 1 | 86 | 7 | 85 | 2 |
|  | 121 | 0.01-1.0 | 0.999 | 11 | 24 | 101 | | 13 | | 90 | 2 | | | 103 | | 3 | 0.005-0.5 | 0.999 | 7 | 17 | 88 | 3 | 94 | 5 | 94 | 13 |
|  | 171 | 0.01-1.0 | 0.999 | -16 | 16 | 99 | | 3 | | 98 | 5 | | | 102 | | 3 | 0.01-1.0 | 0.999 | -3 | 24 | 78 | 11 | 100 | 2 | 90 | 5 |
|  | 150 | 0.005-0.5 | 0.998 | 7 | 22 | 96 | | 14 | | 91 | 15 | | | 86 | | 8 | 0.005-0.5 | 0.999 | 64 | 17 | 75 | 5 | 65 | 5 | 70 | 3 |
|  | 90 | 0.01-1.0 | 0.995 | -16 | 17 | 95 | | 7 | | 84 | 4 | | | 90 | | 9 | 0.005-0.5 | 0.997 | -15 | 19 | 79 | 6 | 89 | 7 | 80 | 6 |
|  | 142 | 0.01-1.0 | 0.999 | -13 | 22 | 91 | | 4 | | 86 | 3 | | | 94 | | 4 | 0.005-0.5 | 0.999 | 19 | 17 | 81 | 1 | 79 | 5 | 80 | 3 |
|  | 181 | 0.02-2.0 | 0.999 | -38 | 23 | 91 | | 13 | | 88 | 2 | | | 95 | | 3 | 0.005-0.5 | 0.999 | -15 | 17 | 84 | 5 | 80 | 5 | 89 | 4 |
|  | 116 | 0.03-3.0 | 0.999 | -12 | 16 | 114 | | 4 | | 98 | 4 | | | 103 | | 6 | 0.005-0.5 | 0.995 | -15 | 27 | 72 | 15 | 97 | 15 | 98 | 5 |
|  | 7 | 0.01-1.0 | 0.999 | 1 | 22 | 94 | | 5 | | 92 | 4 | | | 90 | | 2 | 0.005-1.0 | 0.999 | 47 | 20 | 88 | 5 | 86 | 8 | 96 | 3 |
|  | 22 | 0.01-1.0 | 0.999 | -7 | 20 | 87 | | 11 | | 87 | 3 | | | 95 | | 3 | 0.005-0.5 | 0.999 | -9 | 18 | 90 | 4 | 82 | 6 | 84 | 1 |
|  | 130 | 0.01-1.0 | 0.996 | 11 | 20 | 104 | | 5 | | 96 | 6 | | | 100 | | 6 | 0.005-0.5 | 0.990 | 16 | 23 | 80 | 6 | 90 | 1 | 92 | 9 |
|  | 96 | 0.01-1.0 | 0.999 | -9 | 20 | 92 | | 10 | | 94 | 12 | | | 84 | | 1 | 0.005-0.5 | 0.997 | -1 | 17 | 105 | 5 | 111 | 5 | 114 | 1 |
|  | 148 | 0.01-1.0 | 0.999 | 9 | 17 | 103 | | 7 | | 84 | 2 | | | 102 | | 4 | 0.005-0.5 | 0.998 | 1 | 24 | 94 | 4 | 85 | 2 | 90 | 6 |
|  | 66 | 0.005-0.5 | 0.999 | -5 | 22 | 93 | | 9 | | 106 | 10 | | | 90 | | 7 | 0.005-0.5 | 0.999 | 1 | 22 | 74 | 3 | 75 | 1 | 80 | 3 |
|  | 21 | 0.01-1.0 | 0.999 | -14 | 20 | 94 | | 3 | | 88 | 3 | | | 95 | | 1 | 0.005-0.5 | 0.999 | -3 | 19 | 76 | 3 | 74 | 7 | 80 | 1 |
|  | 144 | 0.01-1.0 | 0.999 | 8 | 20 | 94 | | 0 | | 94 | 2 | | | 110 | | 3 | 0.01-1.0 | 0.998 | 4 | 20 | 79 | 9 | 79 | 8 | 94 | 1 |
|  | 122 | 0.01-1.0 | 0.999 | -12 | 21 | 97 | | 6 | | 93 | 4 | | | 96 | | 2 | 0.005-0.5 | 0.999 | 10 | 28 | 96 | 5 | 86 | 8 | 94 | 2 |
|  | 42 | 0.01-1.0 | 0.999 | 67 | 20 | 92 | | 2 | | 86 | 1 | | | 85 | | 3 | 0.005-0.5 | 0.999 | 26 | 20 | 88 | 2 | 90 | 8 | 91 | 1 |
|  | 64 | 0.005-0.5 | 0.999 | 12 | 18 | 95 | | 1 | | 106 | 1 | | | 100 | | 3 | 0.005-0.5 | 0.999 | 0 | 21 | 92 | 1 | 90 | 9 | 90 | 3 |
|  | 38 | 0.01-1.0 | 0.999 | 15 | 28 | 91 | | 8 | | 90 | 10 | | | 106 | | 10 | 0.01-1.0 | 0.998 | 6 | 15 | 87 | 1 | 84 | 3 | 85 | 13 |
|  | 89 | 0.02-2.0 | 0.999 | 6 | 30 | 104 | | 14 | | 96 | 10 | | | 102 | | 12 | 0.005-0.5 | 0.999 | 3 | 24 | 86 | 4 | 89 | 2 | 94 | 14 |
|  | 61 | 0.01-1.0 | 0.999 | -14 | 17 | 91 | | 1 | | 84 | 2 | | | 90 | | 5 | 0.005-0.5 | 0.996 | -6 | 25 | 80 | 10 | 100 | 3 | 86 | 2 |
|  | 70 | 0.01-1.0 | 0.999 | -13 | 26 | 97 | | 7 | | 94 | 5 | | | 112 | | 12 | 0.005-0.5 | 0.999 | -15 | 25 | 80 | 9 | 89 | 5 | 70 | 11 |
|  | 149 | 0.005-0.5 | 0.999 | 21 | 19 | 95 | | 10 | | 97 | 3 | | | 96 | | 5 | 0.005-0.5 | 0.998 | 48 | 24 | 84 | 5 | 84 | 8 | 76 | 4 |
|  | 145 | 0.005-0.5 | 0.995 | -20 | 22 | 96 | | 15 | | 97 | 3 | | | 96 | | 7 | 0.005-0.5 | 0.999 | -20 | 18 | 80 | 5 | 71 | 2 | 78 | 7 |
|  | 146 | 0.01-1.0 | 0.999 | -9 | 19 | 98 | | 12 | | 92 | 3 | | | 98 | | 3 | 0.005-0.5 | 0.999 | 9 | 24 | 86 | 0 | 86 | 8 | 86 | 4 |
|  | 71 | 0.01-1.0 | 0.999 | 16 | 17 | 91 | | 11 | | 87 | 2 | | | 96 | | 1 | 0.005-0.5 | 0.996 | 28 | 24 | 89 | 6 | 90 | 8 | 80 | 2 |
|  | 210 | 0.01-1.0 | 0.999 | -5 | 16 | 98 | | 3 | | 91 | 1 | | | 94 | | 4 | 0.005-0.5 | 0.995 | 4 | 20 | 87 | 4 | 94 | 4 | 90 | 1 |
|  | 11 | 0.03-1.5 | 0.998 | -12 | 28 | 98 | | 9 | | 112 | 14 | | | 110 | | 18 | 0.005-1.0 | 0.999 | -12 | 18 | 92 | 6 | 80 | 2 | 86 | 13 |
|  | 65 | 0.01-1.0 | 0.999 | 13 | 21 | 106 | | 16 | | 96 | 4 | | | 108 | | 5 | 0.005-0.5 | 0.999 | 15 | 20 | 92 | 3 | 90 | 4 | 86 | 6 |
|  | 189 | 0.01-1.0 | 0.999 | -29 | 25 | 93 | | 8 | | 90 | 5 | | | 82 | | 7 | 0.005-0.5 | 0.999 | 18 | 20 | 90 | 2 | 88 | 4 | 87 | 10 |
|  | 124 | 0.02-2.0 | 0.999 | -10 | 22 | 96 | | 6 | | 89 | 4 | | | 92 | | 5 | 0.005-0.5 | 0.998 | -9 | 19 | 89 | 8 | 73 | 3 | 83 | 7 |
|  | 80 | 0.01-1.0 | 0.995 | 68 | 25 | 106 | | 14 | | 91 | 10 | | | 100 | | 7 | 0.01-1.0 | 0.995 | 23 | 22 | 88 | 5 | 82 | 6 | 91 | 10 |
|  | 178 | 0.01-1.0 | 0.999 | -12 | 20 | 88 | | 4 | | 89 | 2 | | | 88 | | 1 | 0.005-0.5 | 0.999 | 2 | 17 | 84 | 4 | 86 | 1 | 79 | 4 |
|  | 151 | 0.005-0.5 | 0.999 | -10 | 18 | 96 | | 2 | | 90 | 8 | | | 104 | | 11 | 0.005-0.5 | 0.997 | -19 | 18 | 83 | 5 | 87 | 2 | 92 | 9 |
|  | 31 | 0.01-1.0 | 0.997 | 34 | 18 | 96 | | 1 | | 90 | 2 | | | 94 | | 2 | 0.005-0.5 | 0.998 | 5 | 20 | 77 | 8 | 85 | 4 | 93 | 2 |
|  | 211 | 0.01-1.0 | 0.999 | -6 | 20 | 86 | | 15 | | 81 | 25 | | | 100 | | 17 | 0.005-0.5 | 0.999 | -10 | 17 | 90 | 4 | 89 | 1 | 97 | 11 |
|  | 92 | 0.01-1.0 | 0.999 | 26 | 17 | 91 | | 2 | | 85 | 3 | | | 98 | | 10 | 0.005-0.5 | 0.999 | 7 | 16 | 80 | 4 | 81 | 0 | 88 | 1 |
|  | 194 | 0.02-2.0 | 0.999 | -9 | 18 | 90 | | 12 | | 94 | 13 | | | 84 | | 3 | 0.005-0.5 | 0.999 | -2 | 17 | 91 | 1 | 92 | 1 | 90 | 2 |
|  | 24 | 0.03-3.0 | 0.998 | -25 | 21 | 72 | | 10 | | 75 | 1 | | | 83 | | 7 | 0.005-0.5 | 0.999 | 0 | 22 | 78 | 5 | 71 | 6 | 80 | 5 |
|  | 175 | 0.05-5.0 | 0.999 | 32 | 20 | 104 | | 10 | | 96 | 1 | | | 96 | | 5 | 0.01-1.0 | 0.999 | -25 | 22 | 88 | 3 | 89 | 6 | 93 | 4 |
|  | 56 | 0.01-0.5 | 0.999 | 9 | 19 | 104 | | 3 | | 88 | 3 | | | 103 | | 3 | 0.005-0.5 | 0.999 | 11 | 27 | 95 | 5 | 88 | 1 | 85 | 3 |
|  | 68 | 0.01-1.0 | 0.999 | -25 | 18 | 98 | | 11 | | 98 | 4 | | | 101 | | 2 | 0.005-0.5 | 0.998 | -16 | 27 | 76 | 3 | 72 | 1 | 78 | 2 |
|  | 216 | 0.01-1.0 | 1.000 | -12 | 16 | 86 | | 11 | | 78 | 7 | | | 84 | | 10 | 0.005-0.5 | 0.998 | -9 | 27 | 80 | 4 | 88 | 11 | 84 | 7 |
|  | 115 | 0.02-2.0 | 0.995 | -39 | 21 | 80 | | 10 | | 78 | 12 | | | 72 | | 13 | 0.005-0.5 | 0.998 | -25 | 28 | 99 | 12 | 83 | 2 | 75 | 12 |
|  | 20 | 0.01-1.0 | 0.999 | -13 | 19 | 92 | | 3 | | 84 | 3 | | | 95 | | 2 | 0.005-0.5 | 0.997 | -18 | 27 | 90 | 5 | 82 | 1 | 81 | 3 |
|  | 164 | 0.01-1.0 | 0.999 | -15 | 16 | 104 | | 6 | | 94 | 6 | | | 102 | | 6 | 0.005-0.5 | 0.998 | -14 | 26 | 90 | 5 | 80 | 1 | 89 | 7 |
|  | 1 | 0.01-1.0 | 0.999 | -39 | 20 | 97 | | 3 | | 96 | 6 | | | 102 | | 5 | 0.01-1.0 | 0.998 | -1 | 22 | 86 | 4 | 88 | 6 | 93 | 4 |
|  | 203 | 0.01-1.0 | 0.999 | 5 | 20 | 104 | | 5 | | 97 | 12 | | | 108 | | 7 | 0.005-0.5 | 0.998 | 9 | 23 | 83 | 6 | 95 | 7 | 89 | 4 |
|  | 19 | 0.01-1.0 | 0.999 | 12 | 20 | 104 | | 14 | | 94 | 4 | | | 108 | | 4 | 0.005-0.5 | 0.998 | 35 | 18 | 92 | 3 | 93 | 2 | 88 | 4 |
|  | 67 | 0.01-1.0 | 0.999 | -2 | 19 | 90 | | 4 | | 93 | 4 | | | 84 | | 5 | 0.005-0.5 | 0.999 | 12 | 28 | 80 | 10 | 89 | 2 | 100 | 3 |
|  | 28 | 0.01-1.0 | 0.999 | -14 | 18 | 60 | | 1 | | 66 | 2 | | | 68 | | 2 | 0.01-1.0 | 0.999 | -3 | 17 | 70 | 3 | 73 | 1 | 76 | 2 |
|  | 83 | 0.02-2.0 | 0.999 | -10 | 22 | 103 | | 12 | | 96 | 5 | | | 106 | | 5 | 0.005-0.5 | 0.999 | -1 | 23 | 75 | 7 | 89 | 7 | 82 | 6 |
|  | 60 | 0.01-1.0 | 0.999 | 80 | 16 | 106 | | 6 | | 95 | 3 | | | 89 | | 4 | 0.005-0.5 | 0.999 | 57 | 25 | 76 | 5 | 80 | 9 | 85 | 7 |
|  | 29 | 0.02-2.0 | 0.999 | -40 | 26 | 87 | | 4 | | 89 | 2 | | | 82 | | 1 | 0.01-1.0 | 0.999 | -10 | 20 | 89 | 2 | 86 | 4 | 90 | 17 |
|  | 13 | 0.01-1.0 | 0.999 | 13 | 17 | 95 | | 3 | | 93 | 1 | | | 99 | | 2 | 0.005-1.0 | 0.999 | 9 | 17 | 76 | 5 | 80 | 1 | 70 | 1 |
|  | 136 | 0.01-1.0 | 0.999 | -21 | 16 | 94 | | 12 | | 89 | 17 | | | 100 | | 8 | 0.005-0.5 | 0.997 | -18 | 22 | 79 | 3 | 80 | 6 | 84 | 7 |
|  | 12 | 0.02-1.0 | 0.997 | -35 | 18 | 86 | | 2 | | 96 | 2 | | | 82 | | 10 | 0.005-1.0 | 0.997 | -25 | 17 | 76 | 1 | 71 | 1 | 80 | 9 |
|  | 84 | 0.01-1.0 | 0.999 | -7 | 16 | 91 | | 5 | | 86 | 3 | | | 93 | | 3 | 0.005-0.5 | 0.997 | -5 | 26 | 85 | 4 | 92 | 10 | 91 | 2 |
|  | 73 | 0.02-2.0 | 0.999 | -13 | 16 | 88 | | 13 | | 85 | 2 | | | 94 | | 1 | 0.005-0.5 | 0.999 | -3 | 21 | 96 | 5 | 87 | 5 | 97 | 2 |
|  | 132 | 0.01-1.0 | 0.998 | -5 | 18 | 89 | | 6 | | 86 | 4 | | | 96 | | 7 | 0.005-0.5 | 0.998 | 2 | 17 | 90 | 1 | 88 | 1 | 89 | 4 |
|  | 75 | 0.02-2.0 | 0.999 | -27 | 18 | 93 | | 4 | | 88 | 5 | | | 84 | | 7 | 0.005-0.5 | 0.999 | 18 | 20 | 93 | 9 | 87 | 4 | 76 | 4 |
|  | 195 | 0.02-2.0 | 0.999 | -8 | 16 | 94 | | 2 | | 108 | 2 | | | 100 | | 3 | 0.005-0.5 | 0.999 | -4 | 21 | 85 | 8 | 70 | 5 | 77 | 2 |
|  | 77 | 0.01-1.0 | 0.999 | -20 | 20 | 99 | | 14 | | 92 | 5 | | | 96 | | 6 | 0.005-0.5 | 0.999 | -9 | 21 | 80 | 2 | 84 | 5 | 80 | 6 |
|  | 202 | 0.01-1.0 | 0.999 | 11 | 17 | 110 | | 1 | | 94 | 2 | | | 94 | | 2 | 0.005-0.5 | 0.999 | 6 | 19 | 92 | 3 | 86 | 3 | 87 | 3 |
|  | 214 | 0.01-1.0 | 0.999 | 63 | 17 | 92 | | 4 | | 93 | 5 | | | 90 | | 4 | 0.005-0.5 | 0.996 | 1 | 18 | 90 | 2 | 94 | 2 | 91 | 1 |
|  | 9 | 0.01-0.5 | 0.999 | 19 | 22 | 102 | | 9 | | 94 | 9 | | | 102 | | 6 | 0.005-1.0 | 0.997 | 1 | 22 | 88 | 6 | 86 | 6 | 98 | 8 |
|  | 95 | 0.01-1.0 | 0.999 | 4 | 25 | 90 | | 13 | | 99 | 2 | | | 105 | | 9 | 0.005-0.5 | 0.997 | 5 | 19 | 85 | 4 | 78 | 3 | 86 | 11 |
|  | 163 | 0.01-1.0 | 0.999 | -45 | 18 | 98 | | 5 | | 90 | 3 | | | 102 | | 5 | 0.005-0.5 | 0.995 | 22 | 17 | 99 | 8 | 92 | 1 | 84 | 4 |
|  | 186 | 0.01-1.0 | 0.999 | -10 | 18 | 98 | | 2 | | 93 | 4 | | | 106 | | 4 | 0.005-0.5 | 0.999 | 17 | 20 | 94 | 4 | 88 | 4 | 86 | 4 |
|  | 123 | 0.01-1.0 | 0.999 | 26 | 16 | 100 | | 12 | | 95 | 4 | | | 105 | | 5 | 0.005-0.5 | 0.998 | 4 | 25 | 103 | 8 | 118 | 9 | 109 | 2 |
|  | 5 | 0.01-1.0 | 0.999 | 71 | 21 | 69 | | 15 | | 63 | 11 | | | 64 | | 12 | 0.005-1.0 | 0.999 | 1 | 19 | 79 | 4 | 71 | 3 | 75 | 7 |
|  | 3 | 0.02-2.0 | 0.999 | 24 | 23 | 106 | | 12 | | 93 | 9 | | | 95 | | 7 | 0.005-1.0 | 0.999 | 0 | 22 | 89 | 5 | 85 | 6 | 80 | 9 |
|  | 76 | 0.01-1.0 | 0.999 | -4 | 16 | 94 | | 3 | | 90 | 3 | | | 95 | | 3 | 0.005-0.5 | 0.999 | 10 | 19 | 76 | 6 | 78 | 3 | 88 | 2 |
|  | 8 | 0.01-0.5 | 0.999 | 0 | 18 | 90 | | 13 | | 98 | 3 | | | 91 | | 2 | 0.005-1.0 | 0.999 | 16 | 25 | 83 | 7 | 86 | 9 | 96 | 4 |
|  | 183 | 0.01-1.0 | 0.999 | -13 | 17 | 108 | | 1 | | 91 | 3 | | | 95 | | 2 | 0.01-1.0 | 0.996 | 19 | 17 | 99 | 10 | 82 | 1 | 81 | 1 |
|  | 139 | 0.01-1.0 | 0.999 | 10 | 20 | 105 | | 14 | | 96 | 3 | | | 108 | | 4 | 0.005-0.5 | 0.999 | 0 | 17 | 111 | 1 | 109 | 1 | 109 | 6 |
|  | 82 | 0.02-2.0 | 0.999 | 6 | 17 | 80 | | 11 | | 85 | 23 | | | 93 | | 4 | 0.005-0.5 | 0.997 | 1 | 17 | 92 | 3 | 92 | 1 | 86 | 3 |
|  | 14 | 0.02-2.0 | 0.999 | -12 | 17 | 94 | | 1 | | 91 | 1 | | | 82 | | 10 | 0.005-1.0 | 0.999 | -19 | 26 | 86 | 5 | 95 | 10 | 89 | 1 |
|  | 192 | 0.01-1.0 | 0.999 | 18 | 18 | 92 | | 15 | | 90 | 4 | | | 98 | | 5 | 0.005-0.5 | 0.999 | 1 | 26 | 94 | 4 | 87 | 10 | 87 | 4 |
|  | 160 | 0.04-4.0 | 0.999 | 71 | 25 | 70 | | 7 | | 88 | 14 | | | 78 | | 15 | 0.01-1.0 | 0.999 | 3 | 17 | 82 | 4 | 90 | 1 | 86 | 11 |
|  | 184 | 0.02-2.0 | 0.995 | 32 | 18 | 95 | | 1 | | 96 | 2 | | | 90 | | 3 | 0.005-0.5 | 0.998 | 49 | 17 | 89 | 7 | 76 | 1 | 86 | 4 |
|  | 93 | 0.01-1.0 | 0.999 | -13 | 17 | 92 | | 2 | | 90 | 1 | | | 94 | | 1 | 0.005-0.5 | 0.999 | -10 | 18 | 81 | 3 | 80 | 2 | 76 | 1 |
|  | 169 | 0.01-1.0 | 0.999 | -9 | 19 | 94 | | 7 | | 90 | 5 | | | 98 | | 4 | 0.005-0.5 | 0.999 | -1 | 21 | 88 | 2 | 92 | 5 | 90 | 5 |
|  | 78 | 0.01-1.0 | 0.999 | -14 | 16 | 86 | | 12 | | 92 | 11 | | | 86 | | 2 | 0.005-0.5 | 0.999 | -18 | 18 | 82 | 4 | 82 | 2 | 89 | 2 |
|  | 39 | 0.01-1.0 | 0.999 | 12 | 21 | 102 | | 10 | | 91 | 7 | | | 96 | | 7 | 0.01-1.0 | 0.999 | 5 | 24 | 89 | 8 | 76 | 4 | 91 | 7 |
|  | 40 | 0.03-3.0 | 0.999 | 5 | 17 | 101 | | 5 | | 102 | 3 | | | 94 | | 3 | 0.005-0.5 | 0.999 | 1 | 26 | 85 | 4 | 78 | 6 | 86 | 3 |
|  | 15 | 0.02-1.0 | 0.999 | 10 | 16 | 97 | | 11 | | 93 | 1 | | | 94 | | 2 | 0.005-1.0 | 0.999 | 17 | 25 | 96 | 5 | 85 | 9 | 89 | 2 |
|  | 190 | 0.02-2.0 | 0.999 | -9 | 16 | 91 | | 11 | | 89 | 4 | | | 96 | | 1 | 0.005-0.5 | 0.998 | 3 | 27 | 101 | 10 | 84 | 11 | 85 | 2 |
|  | 69 | 0.02-2.0 | 0.999 | 53 | 19 | 101 | | 5 | | 95 | 3 | | | 108 | | 5 | 0.01-1.0 | 0.999 | 0 | 20 | 75 | 3 | 70 | 4 | 75 | 5 |
|  | 182 | 0.03-3.0 | 0.999 | -28 | 23 | 111 | | 17 | | 98 | 8 | | | 98 | | 9 | 0.005-0.5 | 0.996 | -19 | 24 | 83 | 2 | 86 | 8 | 84 | 9 |
|  | 193 | 0.01-1.0 | 0.999 | 29 | 18 | 105 | | 9 | | 115 | 7 | | | 109 | | 14 | 0.01-1.0 | 0.999 | 1 | 22 | 70 | 4 | 76 | 6 | 77 | 4 |
|  | 55 | 0.01-1.0 | 0.999 | -1 | 18 | 91 | | 12 | | 90 | 1 | | | 92 | | 1 | 0.005-0.5 | 0.995 | -10 | 27 | 91 | 5 | 89 | 11 | 81 | 4 |
|  | 45 | 0.02-2.0 | 0.999 | 20 | 18 | 96 | | 7 | | 87 | 3 | | | 88 | | 3 | 0.005-0.5 | 0.999 | 1 | 27 | 120 | 4 | 116 | 2 | 112 | 4 |
|  | 114 | 0.02-2.0 | 0.999 | 7 | 20 | 105 | | 17 | | 93 | 5 | | | 93 | | 7 | 0.005-0.5 | 0.999 | 0 | 18 | 91 | 4 | 84 | 2 | 88 | 6 |
|  | 10 | 0.02-2.0 | 0.999 | -9 | 18 | 96 | | 4 | | 89 | 2 | | | 98 | | 5 | 0.005-1.0 | 0.995 | -1 | 25 | 102 | 3 | 108 | 9 | 108 | 4 |
|  | 74 | 0.02-2.0 | 0.999 | 17 | 19 | 108 | | 4 | | 96 | 3 | | | 100 | | 5 | 0.005-0.5 | 0.999 | 1 | 24 | 88 | 4 | 90 | 4 | 83 | 5 |
|  | 59 | 0.03-3.0 | 0.999 | -10 | 16 | 98 | | 4 | | 94 | 4 | | | 98 | | 4 | 0.005-0.5 | 0.998 | -16 | 20 | 97 | 8 | 84 | 4 | 84 | 2 |
|  | 113 | 0.01-1.0 | 0.999 | -3 | 16 | 96 | | 8 | | 93 | 4 | | | 90 | | 2 | 0.005-0.5 | 0.999 | -2 | 27 | 80 | 3 | 84 | 7 | 85 | 2 |
|  | 174 | 0.03-3.0 | 0.999 | -2 | 20 | 96 | | 18 | | 99 | 3 | | | 104 | | 2 | 0.005-0.5 | 0.999 | -2 | 21 | 112 | 3 | 118 | 5 | 113 | 6 |
